# Pregnane steroidogenesis is altered by HIV-1 Tat and morphine: Physiological allopregnanolone is protective against neurotoxic and psychomotor effects

**DOI:** 10.1016/j.ynstr.2020.100211

**Published:** 2020-01-29

**Authors:** Jason J. Paris, Philippe Liere, Sarah Kim, Fakhri Mahdi, Meagan E. Buchanan, Sara R. Nass, Alaa N. Qrareya, Mohammed F. Salahuddin, Antoine Pianos, Neïké Fernandez, Zia Shariat-Madar, Pamela E. Knapp, Michael Schumacher, Kurt F. Hauser

**Affiliations:** a Department of BioMolecular Sciences, University of Mississippi, School of Pharmacy, University, MS, 38677-1848, USA; b Research Institute of Pharmaceutical Sciences, University of Mississippi, University, MS, 38677, USA; c U1195 Inserm and University Paris-Sud and University Paris-Saclay, 94276, Le Kremlin-Bicêtre, France; d Department of Anatomy and Neurobiology, Virginia Commonwealth University, School of Medicine, Richmond, VA, 23298, USA; e Department of Pharmacology and Toxicology, School of Medicine, Virginia Commonwealth University, Richmond, VA, 23298, USA; f Institute for Drug and Alcohol Studies, Virginia Commonwealth University, Richmond, VA, 23298-0059, USA

**Keywords:** 5α-pregnan-3α-ol-20-one, Glucocorticoid resistance, HIV/AIDS, Opioid, Neurosteroid, Stress

## Abstract

Pregnane steroids, particularly allopregnanolone (AlloP), are neuroprotective in response to central insult. While unexplored *in vivo*, AlloP may confer protection against the neurological dysfunction associated with human immunodeficiency virus type 1 (HIV-1). The HIV-1 regulatory protein, trans-activator of transcription (Tat), is neurotoxic and its expression in mice increases anxiety-like behavior; an effect that can be ameliorated by progesterone, but not when 5α-reduction is blocked. Given that Tat's neurotoxic effects involve mitochondrial dysfunction and can be worsened with opioid exposure, we hypothesized that Tat and/or combined morphine would perturb steroidogenesis in mice, promoting neuronal death, and that exogenous AlloP would rescue these effects. Like other models of neural injury, conditionally inducing HIV-1 Tat in transgenic mice significantly increased the central synthesis of pregnenolone and progesterone's 5α-reduced metabolites, including AlloP, while decreasing central deoxycorticosterone (independent of changes in plasma). Morphine significantly increased brain and plasma concentrations of several steroids (including progesterone, deoxycorticosterone, corticosterone, and their metabolites) likely via activation of the hypothalamic-pituitary-adrenal stress axis. Tat, but not morphine, caused glucocorticoid resistance in primary splenocytes. In neurons, Tat depolarized mitochondrial membrane potential and increased cell death. Physiological concentrations of AlloP (0.1, 1, or 10 nM) reversed these effects. High-concentration AlloP (100 nM) was neurotoxic in combination with morphine. Tat induction in transgenic mice potentiated the psychomotor effects of acute morphine, while exogenous AlloP (1.0 mg/kg, but not 0.5 mg/kg) was ameliorative. Data demonstrate that steroidogenesis is altered by HIV-1 Tat or morphine and that physiological AlloP attenuates resulting neurotoxic and psychomotor effects.

## Highlights

• HIV-1 Tat increases brain pregnenolone and progesterone's 5α-reduced metabolites.
• Morphine increases brain/plasma progesterone, deoxycorticosterone, & corticosterone.
• Tat depolarizes, morphine hyperpolarizes, & AlloP restores, mitochondrial membranes.
• Tat exerts direct neurotoxicity and physiological AlloP attenuates this effect.
• Tat potentiates morphine's psychomotor effects and AlloP attenuates these effects.

## 1 Introduction

Drugs of abuse interact with human immunodeficiency virus type 1 (HIV-1) proteins to promote central nervous system (CNS) dysfunction and/or toxicity in cultured neural cells or animal models (Cai et al., 2016; Mediouni et al., 2015a, Mediouni et al., 2015b; Nath et al., 2002; Silverstein et al., 2012; Soontornniyomkij et al., 2016). In particular, the HIV-1 regulatory protein, trans-activator of transcription (Tat), promotes neuroinflammation via the activation of infected and uninfected microglia and astrocytes, indirectly contributing to neurotoxicity (Hauser et al., 2006; Nath et al., 2002). Opioids such as morphine, heroin, methadone, and buprenorphine can exacerbate Tat-mediated cytokine production (Festa and Meucci, 2012; Fitting et al., 2014a; Hauser et al., 2012; Nath et al., 2002). However, Tat is also directly neurotoxic via multiple mechanisms including the activation of NMDA receptors, voltage-gated L-type Ca^2+^ channels, and mitochondrial dysfunction, resulting in bioenergetic crisis and cell death (Chopard et al., 2018; Eugenin et al., 2007; Haughey et al., 2001; Hategan et al., 2017; Krogh et al., 2014; Lecoeur et al., 2012; Malik et al., 2011; Napier et al., 2014; Spector et al., 2019). The influence of opioids on Tat's direct, neurotoxic effects are not well-understood, but may involve mitochondrial-mediated processes including steroidogenesis.

Despite treatment with combined antiretroviral therapeutics, a considerable proportion of patients infected with HIV-1 present with endocrine dysfunction. Approximately 25% of HIV^+^ patients experience dysfunction of the hypothalamic-pituitary-gonadal (HPG; e.g., hypogonadism) and/or hypothalamic-pituitary-adrenal (HPA) axes (e.g., elevated basal corticosterone with adrenal insufficiency in response to HPA activation; Chrousos and Zapanti, 2014; George and Bhangoo, 2013; Gomes et al., 2017; Lachâtre et al., 2017; Mirza et al., 2018; Wong et al., 2017). In the era of combined antiretroviral therapy, HPG and HPA disruption typically involve dysfunction within the CNS, rather than dysfunction at peripheral steroid sources such as the gonads or adrenals (Bons et al., 2013; Chrousos and Zapanti, 2014; Freda and Bilezikian, 1999; Rochira and Guaraldi, 2014; Mirza et al., 2018). In support, others have found evidence of neurosteroidogenic dysregulation in post-mortem HIV^+^ brain tissue, cultured human fetal neurons, and the brains of cats infected with feline immunodeficiency virus (Maingat et al., 2013). Maintaining HPG/HPA function may improve neurological outcomes.

Restoration of physiological steroid content may ameliorate neurological deficits caused by HIV-1 Tat and/or combined opioid exposure. Estradiol or pregnane steroids [progesterone (PROG) or the 3α-hydroxy/5α-reduced PROG metabolite, allopregnanolone (AlloP)] rescue Tat-mediated neurotoxicity *in vitro* (Adams et al., 2010, 2012; Kendall et al., 2005; Paris et al., 2016; Salahuddin et al., 2019; Wallace et al., 2006). Moreover, transgenic expression of HIV-1 Tat in male or female mice increases anxiety- and depression-like behavior *in vivo* (McLaughlin et al., 2017; Paris et al., 2014d; Salahuddin et al., 2019). In females, these effects coincide with Tat-mediated elevations in circulating corticosterone and are exacerbated by exposure to the clinically-used opioid, oxycodone (Salahuddin et al., 2019). Exogenous PROG can ameliorate Tat-mediated anxiety-like effects in mice (Paris et al., 2014c, 2016) and this appears to be dependent on metabolism to AlloP (Paris et al., 2016). Thus, the PROG metabolite, AlloP, may confer protection against the CNS-targeted effects of HIV-1 Tat, but additional evidence is needed to confirm this and to assess its effects on Tat and opioid interactions.

Given that mitochondrial function is both a target for HIV-1 Tat and a rate-limiting step for neurosteroidogenesis to occur, we hypothesized that conditional expression of an HIV-1 *tat* transgene in mice would alter central and/or circulating steroid content and that morphine would further influence these effects. We expected this to co-occur with neuronal mitochondrial dysfunction and neuron cell death. Lastly, we anticipated the pregnane steroid, AlloP (previously found to exert neuroprotection in cell culture), would ameliorate Tat-mediated mitochondrial dysfunction, cell death, and the potentiation of morphine's behavioral effects.

## 2 Materials and methods

The use of mice in these studies was pre-approved by the Institutional Animal Care and Use Committees at Virginia Commonwealth University and the University of Mississippi. All experiments were conducted in accordance with ethical guidelines defined by the National Institutes of Health (NIH Publication No. 85-23).

### 2.1 Animals and housing

Steroid analysis and behavioral studies utilized adult male mice that expressed (or did not express) a GFAP-driven, doxycycline-inducible, HIV–1_IIIB_ *tat*_1-86_ transgene (N = 355) as previously described (Bruce-Keller et al., 2008). Tat(−) and Tat(+) mice were generated in the vivaria at Virginia Commonwealth University (MCV campus; for steroid analyses and glucocorticoid insensitivity assay) and at the University of Mississippi (for behavioral analyses). Primary cell culture analyses utilized offspring acquired from timed-mated, C57BL/6J females (N = 10; obtained from the Jackson Laboratory, Bar Harbor, ME). All mice were ~70 days of age at the time of testing, were housed 4–5/cage (Tat-transgenic mice) or were singly-housed (timed-mated C57BL/6J mice), and were maintained in a temperature- and humidity-controlled room on a 12:12 h light/dark cycle with *ad libitum* access to food and water.

### 2.2 Chemicals

#### 2.2.1 Chemicals used *in vitro*

Cells were treated with vehicle or a saturating dose of morphine (500 nM in ddH_2_0; #M8777, Sigma-Aldrich, Saint Louis, MO), vehicle or low-to-high AlloP (0.1, 1, 10, or 100 nM dissolved in DMSO and diluted 1:10,000 in media; #P8887, Sigma-Aldrich), and vehicle or HIV-1 Tat_1-86_ (100 nM diluted to concentration in ddH_2_0; #1002-2, ImmunoDx, Woburn, MA). AlloP dosing reflects a range of low-to-high physiological concentrations that have been found to confer protection from several neurodegenerative insults (Ardeshiri et al., 2006; Irwin and Brinton, 2014; Irwin et al., 2015; Lockhart et al., 2002; Waters et al., 1997). The concentration of Tat reflects one from a range that elicits functional deficits in glia and neurons similar to those observed in HIV infection (Kruman et al., 1998; Nath et al., 1999; Singh et al., 2004; El-Hage et al., 2005, 2008; Perry et al., 2010). Splenocytes were treated with lipopolysaccharide (LPS) O26:B6 (204 nM in PBS; #L3755, Sigma-Aldrich) and vehicle or low-to-high corticosterone (0.005, 0.05, 0.1, 0.5, or 5 μM) dissolved in EtOH and diluted 1:25 in media (#27840, Sigma-Aldrich). SH-SY5Y cells were differentiated via sequential exposure to retinoic acid (1.5 mg/ml dissolved in 95% EtOH and protected from light; #R2625, Sigma-Aldrich) and BDNF (10 μg/ml dissolved in DMEM/F12; #SRP3014, Sigma-Aldrich) as described below.

#### 2.2.2 Chemicals used *in vivo*

Mice were treated with vehicle (saline 0.9%) or morphine (30 mg/kg, i.p.; #M8777, Sigma-Aldrich) as well as vehicle or AlloP (0.5 or 1 mg/kg, s.c., dissolved in EtOH and diluted 1:10 in oil; #P8887, Sigma-Aldrich). To induce HIV-1 *tat*_*1-86*_ transgene expression (or not), Tat(+) and Tat(−) control mice were placed on doxycycline chow (6 g/kg; Dox Diet #2018, Harlan Laboratories, Madison, WI) for 4 weeks in order to assess changes in steroidogenesis. To assess the influence of Tat and morphine on glucocorticoid insensitivity, Tat transgene expression was induced for 8 weeks by doxycycline chow, and mice were administered saline or ramping doses of morphine (10–40 mg/kg, s.c. BID; increasing by 10 mg/kg every 2 days). To assess the combined influence of Tat and AlloP on morphine-mediated psychomotor behavior, transgene expression was induced via injection of doxycycline (30 mg/kg, i.p.; #14422, Cayman Chemical, Ann Arbor, MI) for 5 days with an additional two days of doxycycline washout prior to behavioral testing (to minimize any potential non-specific behavioral effects of doxycycline). Systemic AlloP (0.5 or 1 mg/kg, s.c.) was concurrently-administered for the full duration of the behavioral testing paradigm (8 days in total). AlloP dosing fell within a range that is expected to produce physiological concentrations in the brain (Gabriel et al., 2004) and has previously demonstrated stepwise, dose-dependent changes on affective behavior (Reddy and Kulkarni, 1996). The chosen morphine concentration is found to enhance AlloP in the rodent brain and circulation when administered acutely (Concas et al., 2006; Porcu et al., 2014).

### 2.3 Cell culture

#### 2.3.1 Differentiated human neuroblastoma cells (SH-SY5Y)

SH-SY5Y neuroblastoma cells were obtained from ATCC (#CRL-2266, Manassas, VA). These cells were chosen to assess the direct neurotoxic effects of HIV-1 Tat on neurons given that they (1) are devoid of glial inputs (obviating Tat's indirect neurotoxic contributions via glially-mediated neuroinflammation), (2) express the endocrine enzymes/receptors and opioid receptors of interest (Grassi et al., 2013; Melcangi et al., 1993; Saify and Saadat, 2016; Salahuddin et al., 2019; Toll et al., 1997), and (3) have been used for similar work previously (Cai and Cadet, 2008; Hong et al., 2004; Hu et al., 2012; Mastrantonio et al., 2016; Sun et al., 2016; Zhu et al., 2009). Cells were seeded onto 96-well plates at a density of 5 × 10^3^/well for assessment of mitochondrial membrane potential or were seeded onto 24-well plates at a density of 2.5 × 10^4^/well for assessment of cell death. Prior to differentiation, cells were maintained in growth media: 89.5% DMEM/F12 (#11320-033, Life Technologies, Carlsbad, CA), 10% heat-inactivated fetal bovine serum (FBS; #SH30071.03, Thermo Scientific Hyclone, Logan, UT), and 0.5% antibiotic/antimycotic mixture (#15240-062, Life Technologies). One day after seeding, growth media was fully exchanged for differentiation media #1 which contained retinoic acid diluted 1:500 in growth media. After 5 d in differentiation media #1, media was fully exchanged for the serum-free differentiation media #2 consisting of BDNF diluted 1:200 in DMEM/F12 (supplemented only with the 0.5% antibiotic/antimycotic mixture). Cells underwent experimental manipulation 3 d later. Throughout differentiation, media was exchanged every 48 h. Differentiation with these factors promotes cell cycle arrest, expression of mature neuron markers (including a shift from nestin^+^ to microtubule-associated protein 2^+^ expression), and a polarized morphology (Constantinescu et al., 2007; Encinas et al., 2000; Murillo et al., 2017; Salahuddin et al., 2019; Shipley et al., 2016).

#### 2.3.2 Primary C57BL/6J striatal neurons

Primary striatal medium spiny neuron cultures were prepared as previously described (Kim et al., 2018; Zou et al., 2011). These cells were utilized given prior work demonstrating the dorsal striatum to be among the largest reservoirs of HIV among post-mortem patients (Nath, 2015) and for neurons within this region to be selectively vulnerable to Tat-mediated disruption in mice (Schier et al., 2017). In brief, primary neurons were derived from the striatum of E15-17 C57BL/6J mice. Dissected striata were minced and incubated at 37 °C (5% CO_2_) for 30 min with trypsin (2.5 mg/ml; #T4799, Sigma-Aldrich) and DNase (15 μg/ml; D5025, Sigma-Aldrich) in neurobasal media (#21103049; Life Technologies), supplemented with B-27 (#12587010, Life Technologies), L-glutamine (9.5 mM; Life Technologies), glutamate (25 μM; Sigma-Aldrich), and antibiotic/antimycotic mixture (Life Technologies). Cells were centrifuged, triturated, and twice filtered through a 70 μm pore nylon mesh (Greiner Bio-One) and seeded onto poly-L-lysine (#P2636, Sigma Aldrich) coated 24-well plates (7.5 × 10^4^/well) for 7–8 days in supplemented neurobasal media before experimental manipulation.

#### 2.3.3 Primary Tat-transgenic mouse splenocytes

Primary splenocyte cultures were prepared as previously described (Avitsur et al., 2001; Kinsey et al., 2007) and were used to assess the ability of prolonged HIV-1 Tat and morphine to alter glucocorticoid-induced inhibition of peripheral immune cell viability. Briefly, mice were sacrificed 24 h after administration of the final dose of morphine (or vehicle) and spleens were harvested. Tissues were passed through a 70 μm pore nylon mesh (Greiner Bio-One) with 10 mL of RPMI media (#22400089, Life Technologies) to prepare a cell suspension. Cells were lysed with a hypotonic salt solution (0.16 M NH_4_Cl, 10 mM KHCO_3_, 0.13 mM EDTA) and suspended in RPMI media (#22400089, Life Technologies), supplemented with 10% heat-inactivated fetal bovine serum (FBS; #SH30071.03, Thermo Scientific), 7.5% NaHCO_3_ (#25080094, Life Technologies), and antibiotic/antimycotic mixture (Life Technologies), centrifuged, and filtered through a 70 μm pore nylon mesh (Greiner Bio-One). Duplicate splenocyte samples were seeded onto 96-well plates with corticosterone and stimulated with LPS for 48 h.

### 2.4 Steroid profiling by gas chromatography-tandem mass spectrometry (GC/MS/MS)

Brain and plasma steroid content was measured in Tat(−) and Tat(+) male mice after one month on doxycycline following handling (baseline), saline injection (0.9%, i.p., 60 min post-treatment), or morphine injection (30 mg/kg, i.p., 60 min post-treatment).

#### 2.4.1 Tissue preparation

Blood was collected in heparinized plastic tubes, centrifuged, and plasma (26–168 μl) was collected and stored at −80 °C. Whole brains were harvested, hemisected (mid-sagittally), weighed (141–256 mg), frozen in liquid nitrogen, and stored at −80 °C.

#### 2.4.2 GC/MS/MS

A panel of steroids was identified and quantified simultaneously in individual tissues by GC/MS/MS as previously described (Liere et al., 2000; Zhu et al., 2017). Briefly, steroids were first extracted from hemisected whole brains and plasma with 10 vol of methanol (MeOH) and the following internal standards were added to the extracts for steroid quantification: 2 ng of ^2^H_6_-5α-dihydroprogesterone (DHP; CDN Isotopes, Sainte Foy la Grande, France) for 5α/β-DHP, 2 ng of epietiocholanolone for pregnenolone (PREG), 20α-dihydropregnenolone (20α-DHPREG), 3α/β5α/β-tetrahydroprogesterone (THP), 5α20α-THP, 3α/β5α/β20α-hexahydroprogesterone (HHP) and 3α/β5α/β-tetrahydrodeoxycorticosterone (THDOC), 2 ng of ^13^C_3_-progesterone (P) for P, 2 ng of 19-norPROG for 20α-DHP, 16α-hydroxyprogesterone (16α-OHP), 17α-hydroxyprogesterone (17αOHP), 5α/β-DHDOC (dihydrodeoxycorticosterone), 5 ng of ^13^C_3_-deoxycorticosterone (DOC) for DOC, 10 ng of ^2^H_8_-corticosterone for corticosterone, 5α-dihydrocorticosterone (5α-DHB) and 3α/β5α/β-tetrahydrocorticosterone (THB). Samples were purified and fractionated by solid-phase extraction with the recycling procedure (Liere et al., 2004). Briefly, the extracts were dissolved in 1 ml MeOH and applied to the C18 cartridge (500 mg, 6 ml, International Sorbent Technology, IST), followed by 5 ml of MeOH/H_2_O (85/15, vol/vol). The flow-through, containing the free steroids, was collected and dried. After a previous re-conditioning of the same cartridge with 5 ml H_2_O, the dried samples were dissolved in MeOH/H_2_O (2/8, vol/vol) and re-applied. The cartridge was then washed with 5 ml H_2_O and 5 ml MeOH/H_2_O (1/1, vol/vol) and unconjugated steroids were eluted with 5 ml MeOH/H_2_O (9/1, vol/vol). The fraction containing the unconjugated steroids was then filtered and further purified and fractionated by high-performance liquid chromatography (HPLC). The HPLC system is composed of a WPS-3000SL analytical autosampler and a LPG-3400SD quaternary pump gradient coupled with a SR-3000 fraction collector (Thermo Scientific). The HPLC separation was achieved with a Lichrosorb Diol column (25 cm, 4.6 mm, 5 μm) in a thermostated block at 30 °C. The column was equilibrated in a solvent system of 90% heptane and 10% of a mixture composed of heptane/isopropanol (85/15, vol/vol). Elution was performed at a flow-rate of 1 ml/min, first 90% heptane and 10% of heptane/isopropanol (85/15, vol/vol) for 15 min, then with a linear gradient to 100% acetone in 2 min. The column was washed with acetone for 15 min. Two fractions were collected from the HPLC system: 5α/β-DHP were eluted in the first HPLC fraction (3–15 min) and was next silylated with 50 μl of a mixture N-methyl-N-trimethylsilyltrifluoroacetamide/ammonium iodide/dithioerythritol (1000:2:5 vol/wt/wt) for 15 min at 70 °C. The second fraction (15–29 min) containing PREG and its derivatives, P, DOC, corticosterone and their precursors and their reduced and hydroxylated metabolites were derivatized with 25 μl of HFBA and 25 μl of anhydrous acetone for 1 h at 20 °C. Both fractions were dried under a stream of nitrogen and resuspended in heptane. GC/MS/MS analysis of the biological extracts was performed using an AI 1310 autosampler, a Trace 1310 gas chromatograph (GC), and a TSQ 8000 mass spectrometer (MS) (Thermo Scientific). Injection was performed in the splitless mode at 250 °C (1 min of splitless time) and the temperature of the gas chromatograph oven was initially maintained at 80 °C for 1 min and ramped between 80 and 200 °C at 20 °C/min, then ramped to 300 °C at 5 °C/min and finally ramped to 350 °C at 30 °C/min. The helium carrier gas flow was maintained constant at 1 ml/min during the analysis. The transfer line and ionization chamber temperatures were 300 °C and 200 °C, respectively. Electron impact ionization was used for mass spectrometry with ionization energy of 70 eV and GC/MS/MS analyses were performed in multiple reaction monitoring (MRM) mode with Argon as the collision gas. GC/MS/MS signals were evaluated using a computer workstation by means of the software Excalibur®, release 3.0 (Thermo Scientific). Identification of steroids was supported by their retention time and two or three transitions and quantification was performed according to the more abundant transition with a previously established calibration curve. The range of the limit of detection was roughly 0.5–20 pg according to the steroid structure. The GC/MS/MS analytical procedure was fully validated in terms of accuracy, reproducibility and linearity in mouse brains (Liere et al., 2000; Zhu et al., 2017). Concentrations of 5β-DHP, 5β-DHDOC, 3α,5β-THDOC, and 3α,5β-THB were below the limit of quantification in all samples.

### 2.5 Splenocyte proliferation assay

Glucocorticoid insensitivity was assessed using a *CellTiter 96®AQ*_*ueous*_ *Non-Radioactive Cell Proliferation Assay* kit (#G5421, Promega, Madison, WI) per manufacturer instructions. Briefly, the proliferation assay was performed 48 h after primary Tat(−) and Tat(+) splenocytes were seeded on 96-well plates in duplicate and incubated with LPS and corticosterone. Media was replaced with the tetrazolium (MTS) and phenazine methosulfate (PMS) solution (1:20) and incubated (37 °C; 5% CO_2_) for 4 h and read at 490 nm using a PHERAstar FS microplate reader (BMG Labtech, Cary, NC). Viability of each sample was expressed as the mean optical density (OD) of duplicates. Corticosterone resistance was assessed by calculating the percentage of viable cells in each sample treated with a given dose of corticosterone compared with the same sample treated with vehicle [(OD of corticosterone treated sample/OD of non-treated sample) * 100].

### 2.6 Mitochondrial membrane potential (Δψ_m_)

Mitochondrial membrane potential (Δψ_m_) was assessed using a JC-10 kit per manufacturer recommendations (#22800, AAT Bioquest, Sunnyvale, CA). Briefly, JC-10 (a JC-1 analogue with improved H_2_O solubility) reversibly forms aggregates when Δψ_m_ is maintained; when depolarized, JC-10 falls into its monomeric state. Excited JC-10 aggregates and monomers display distinct emission spectra allowing for a ratiometric assessment of Δψ_m_ (ex/em_aggregate_: 490/590 nm; ex/em_monomer_: 490/525 nm). Experimental treatments were applied to differentiated SH-SY5Y cells seeded on 96-well plates or primary, striatal, medium spiny neurons seeded on 24 well plates and incubated (37 °C; 5% CO_2_) for 30 min, followed by the addition of JC-10 and another incubation (37 °C; 5% CO_2_) of 30 min in the dark. Fluorescence was assessed in SH-SY5Y cells using a PHERAstar FS microplate reader (BMG Labtech) at 5, 15, and 30 min later. Primary neurons were imaged 15 min later on a Zeiss Axio Observer Z1 microscope with Zeiss Axiovision 4.8 software (Carl Zeiss). For both methods, each plate represented one observation (n) in statistical analyses. 96-well plates had four technical replicates per treatment group and 24-well plates had two technical replicates. For imaging, fields within each well were randomly chosen and at least 200 cells were counted per condition. Δψ_m_ was calculated as the monomer:aggregate ratio (presented as percent change from control values).

### 2.7 Live/dead assay

Neuron viability was assessed using a LIVE/DEAD® Viability/Cytotoxicity Kit (#L-7013, Molecular Probes, Eugene, OR) per manufacturer instructions and as described (Salahuddin et al., 2019). Briefly, experimental treatments were applied to primary medium spiny neurons or SH-SY5Y cells seeded on 24-well plates and live/dead assay was performed 20 h later. Prior work utilizing time-lapse microscopy (0–60 h) identified the 20 h time-point as the earliest time when Tat- and AlloP-treated cells significantly diverged on the measure of viability (Paris et al., 2016). A working solution of DEAD Red (ethidium homodimer-2; ex/em: 535/624 nm) and SYTO 10 (ex/em: 495/520 nm) was prepared by diluting stocks in Hank's Balanced Salt Solution (HBSS; 1:500 dilution). Cell media was replaced with the DEAD Red/SYTO 10 working solution and incubated (37 °C; 5% CO_2_) for 15 min in the dark. Cells were washed 3 times with HBSS, fixed with 4% paraformaldehyde, and imaged as described for 24-well plates above. Viability was assessed by calculating the proportion of necrotic cells [# DEAD Red^+^ cells/# Total Cells) * 100].

### 2.8 Locomotor activity

Mice were acclimated to the testing room with 70 dB white noise for 30 min prior to drug administration. Mice were administered saline (0.9%, i.p.) or morphine (30 mg/kg, i.p.) and, 30 min later, were allowed to freely explore an open field apparatus (40 cm × 40 cm × 40 cm; Stoelting Co., Wood Dale, IL) for 5 min. Behavior was tracked and digitally-encoded via ANY-maze animal tracking software (Stoelting Co.). Horizontal and vertical (i.e., rearing) locomotor behaviors were assessed as indices of psychomotor activity (Paris et al., 2016; Salahuddin et al., 2019).

### 2.9 Statistical analyses

**Fig. 1:**
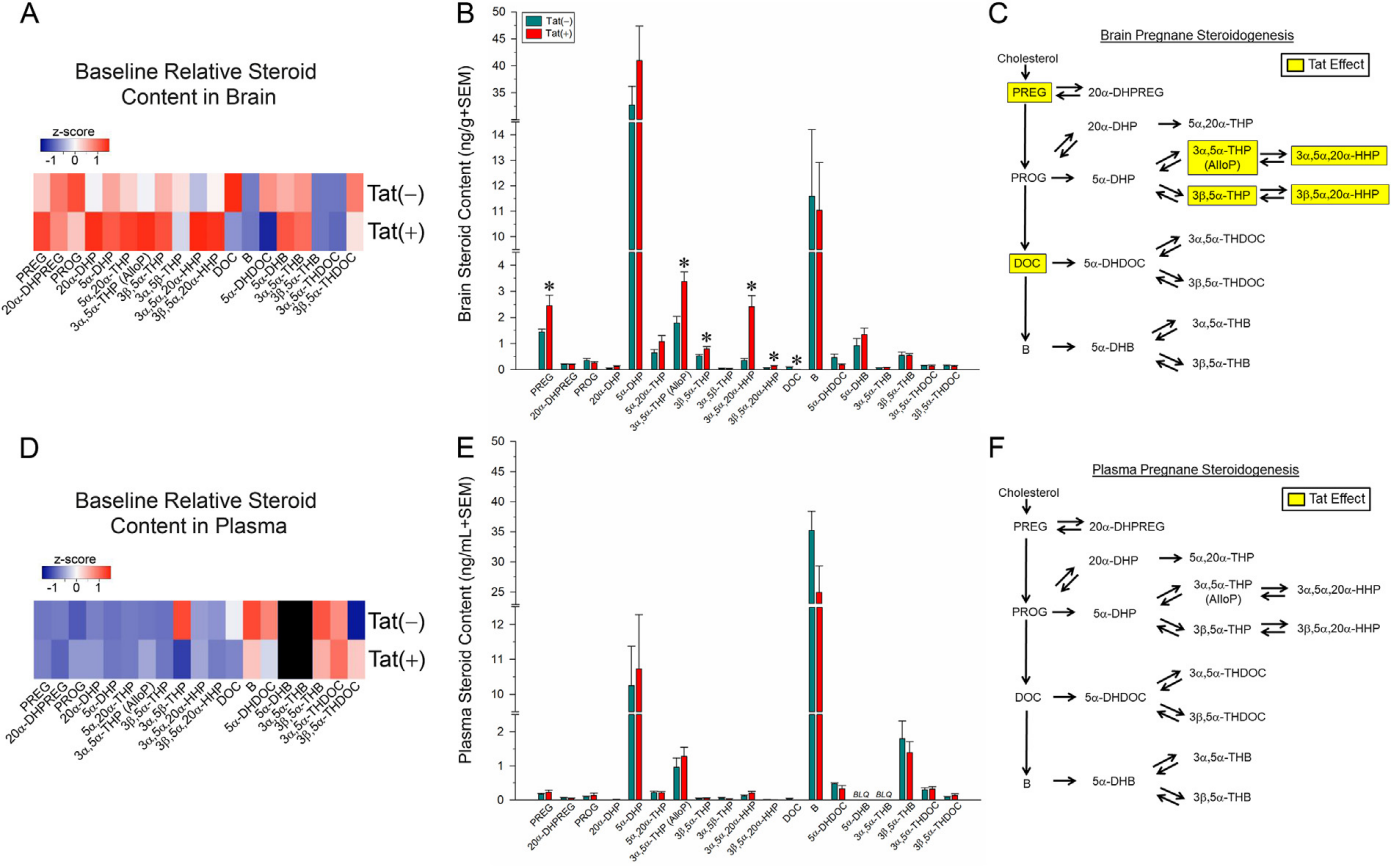
Pregnane steroid content of HIV-1 Tat-transgenic mice [Tat(+)] and non-Tat-expressing controls [Tat(−)] (n = 8/group) was assessed in brain (heatmap in panel A and raw concentrations in panel B) and plasma (heatmap in panel D and raw concentrations in panel E). Tat-mediated steroid changes in the brain are summarized in panel C. Tat expression did not influence steroid content in plasma (see panel F). Black heatmap panels indicate BLQ steroids. * indicates a significant difference between Tat(−) and Tat(+) mice (Student's *t*-test; *p* < 0.05). BLQ = below limit of quantification; DHB = dihydrocorticosterone; DHDOC = dihydrodeoxycorticosterone; DHP = dihydroprogesterone; DHPREG = dihydropregnenolone; DOC = deoxycorticosterone; HHP = hexahydroprogesterone; PREG = pregnenolone; PROG = progesterone; THB = tetrahydrocorticosterone; THDOC = tetrahydrodeoxycorticosterone; THP = tetrahydroprogesterone.

**Fig. 2:**
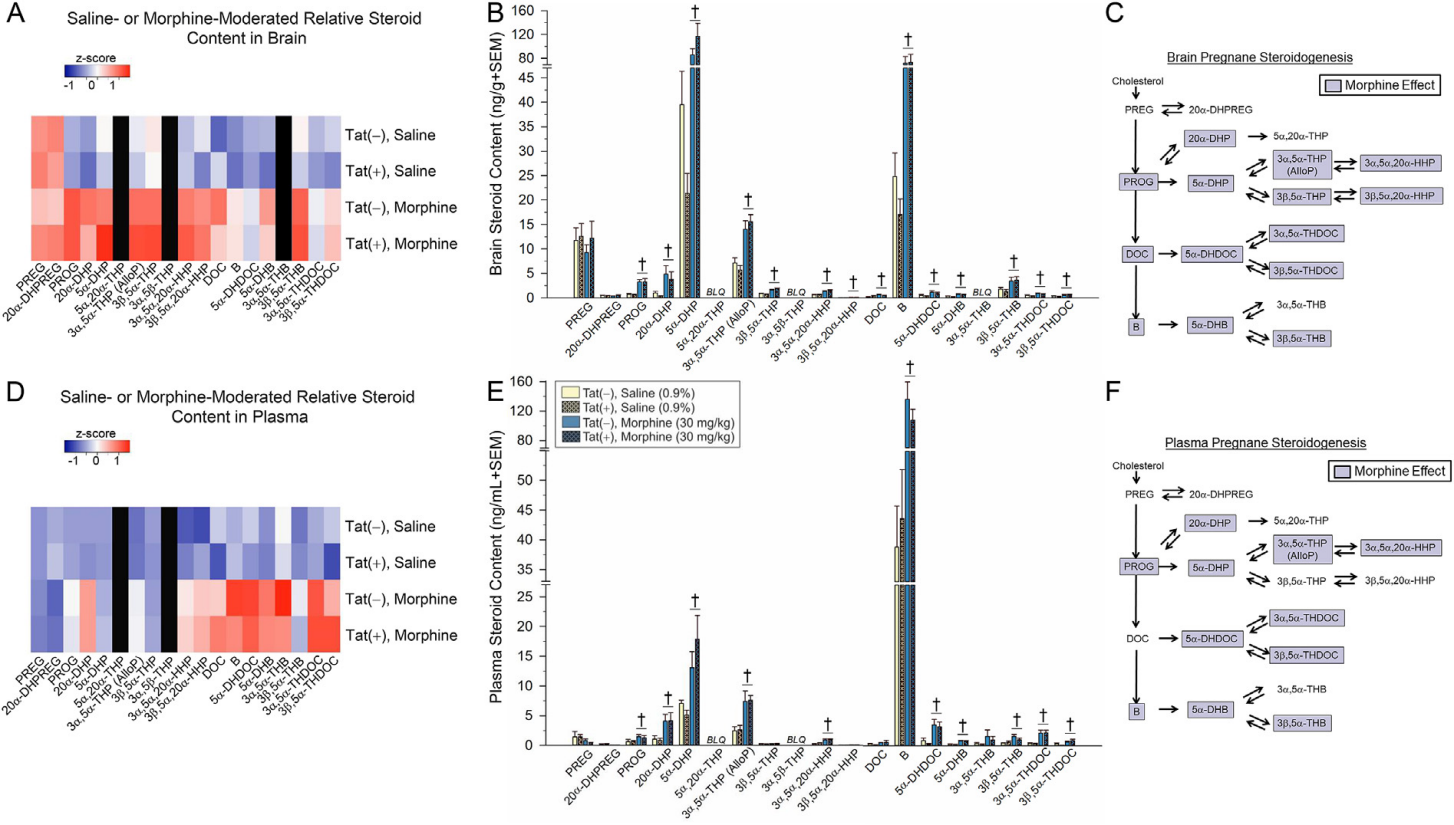
Pregnane steroid content of HIV-1 Tat-transgenic mice [Tat(+)] and non-Tat-expressing controls [Tat(−)] that were administered saline (0.9%, i.p.) or morphine (30 mg/kg, i.p.; n = 8/group), was assessed in brain (heatmap in panel A and raw concentrations in panel B) and plasma (heatmap in panel D and raw concentrations in panel E). Morphine-mediated steroid changes in the brain are summarized in panel C and changes in plasma are summarized in panel F. Black heatmap panels indicate BLQ steroids. † indicates a significant difference between saline- and morphine-treated mice, irrespective of Tat expression (two-way ANOVA; *p* < 0.05). BLQ = below limit of quantification; DHB = dihydrocorticosterone; DHDOC = dihydrodeoxycorticosterone; DHP = dihydroprogesterone; DHPREG = dihydropregnenolone; DOC = deoxycorticosterone; HHP = hexahydroprogesterone; PREG = pregnenolone; PROG = progesterone; THB = tetrahydrocorticosterone; THDOC = tetrahydrodeoxycorticosterone; THP = tetrahydroprogesterone.

All datasets were determined to be normally-distributed with equal variance per Kolmogorov-Smirnov and subsequent Bartlett tests, respectively. Steroid content for each analyte was assessed via separate Student's *t*-tests (Fig. 1) or two-way ANOVAs (Fig. 2) with drug manipulation (saline or morphine) and genotype [(Tat(−) or Tat(+)] as between-subjects factors. Mitochondrial membrane potential across time in SH-SY5Y cells was assessed via repeated measures ANOVA with treatment condition as the between-subjects factors and time as the within-subjects factor. Glucocorticoid insensitivity assays were analyzed via three-way ANOVAs with drug condition (vehicle or morphine), Tat condition (vehicle or Tat), and corticosterone concentration as factors. Remaining analyses for Δψ_m_, live/dead analyses, and behavioral dependent measures were assessed via three-way ANOVAs with drug condition (vehicle- or morphine-exposed), AlloP condition (vehicle- or AlloP-exposed), and Tat condition (control or Tat-exposed) as factors. Fisher's Protected Least Significant Difference *post-hoc* tests determined group differences following main effects. Interactions were delineated via simple main effects and main effect contrasts with alpha controlled for multiple comparisons. For *post-hoc* contrasts, all possible pairwise comparisons were made with the exception of repeated measures analyses conducted in Fig. 4B, wherein *a priori* planned contrasts where performed between all groups and control- (hatched line) or Tat- (red open circle) treatment groups following a significant interaction. All analyses were considered significant when *p* < 0.05.

## 3 Results

### 3.1 HIV-1 Tat alters central steroid biosynthesis

To first assess whether HIV-1 Tat influences pregnane steroidogenesis, central and circulating steroid levels were measured in Tat(−) and Tat(+) male mice. The induction of HIV-1 Tat significantly altered baseline steroid content in the brain (Fig. 1A–C), independent of any effects on circulating steroid content (Fig. 1D–F). Compared to Tat(−) controls, brains collected from Tat(+) mice had significantly greater concentrations of the prohormone, pregnenolone (*p* = 0.03), as well as the 5α-reduced progesterone metabolites, 3α,5α-THP (i.e., AlloP; *p* = 0.003) and 3β,5α-THP (*p* = 0.02), and their respective 20α-reduced metabolites, 3α,5α,20α-HHP (*p* = 0.0003) and 3β,5α,20α-HHP (*p* = 0.01; Fig. 1B). The glucocorticoid precursor, deoxycorticosterone (DOC), was significantly reduced in Tat(+) brains compared to their Tat(−) counterparts (*p* = 0.006; Fig. 1B). No additional baseline differences were observed in pregnane steroid content within the brain (Fig. 1A–C), nor were differences in plasma content of any steroid observed (Fig. 1D–F). Baseline concentrations of 3α,5β,20α-HHP were too low to depict in Fig. 1, but did not change [Tat(−)_Brain_ = 0.008 ± 0.001; Tat(+)_Brain_ = 0.011 ± 0.004; Tat(−)_Plasma_ = 0.004 ± 0.001; Tat(+)_Plasma_ = 0.005 ± 0.001]. Thus, the induction of HIV-1 Tat was associated with increased content of pregnenolone and progesterone's 5α-reduced metabolites, and decreased deoxycorticosterone in the brain (independent of changes in plasma steroid concentrations).

### 3.2 Morphine increases central and peripheral steroid content

In order to assess the additive influence of morphine administration on Tat-mediated steroid biosynthesis, central and circulating steroid contents were measured in Tat(−) and Tat(+) male mice 60 min after an acute injection of saline (0.9%, i.p.) or morphine (30 mg/kg, i.p.). Irrespective of Tat exposure, acute morphine significantly increased brain pregnane steroid biosynthesis, apparently activating the HPA stress axis (Fig. 2A–C). Compared to saline-administration, morphine significantly increased central progesterone (*p* < 0.0001), and its 20α- or 5α-reduced metabolites including 20α-DHP (*p* = 0.0003), 5α-DHP (*p* < 0.0001), AlloP (*p* < 0.0001), 3β,5α-THP (*p* < 0.0001), 3α,5α,20α-HHP (*p* < 0.0009), and 3β,5α,20α-HHP (*p* = 0.008; Fig. 2A–C). Morphine significantly increased all corticosteroids examined in brain compared to saline-induced concentrations (*p* < 0.0001–0.03; Fig. 2A–C) with the exception of 3α,5α-THB, 3α,5β-THB, 5β-DHDOC, and 3α,5β-THDOC which were below the limits of detection in brain (Fig. 2B). 3α,5β,20α-HHP was also below the limit of quantification in brain.

In plasma, acute morphine significantly increased concentrations of progesterone (*p* = 0.008), its 5α-reduced metabolites, 5α-DHP (*p* < 0.0001) and AlloP (*p* < 0.0001), as well as its 20α-reduced metabolites including 20α-DHP (*p* = 0.0003) and 3α,5α,20α-HHP (*p* < 0.0001). As observed in the brain, morphine significantly increased circulating concentrations of all detectable corticosteroids compared to saline-induced content (*p* < 0.0001–0.03; Fig. 2D–F) with the exception of 3α,5α-THB which only tended to be increased (*p* = 0.059, *n.s.*). Circulating pregnenolone and 20α-DHPREG did not differ from saline administration. 3α,5β,20α-HHP was below the limit of quantification in plasma. Thus, acute morphine administration activated the HPA axis, increasing pregnane and corticosteroid contents in brain and plasma.

### 3.3 Exposure to HIV-1 Tat, but not morphine, induces glucocorticoid resistance

**Fig. 3:**
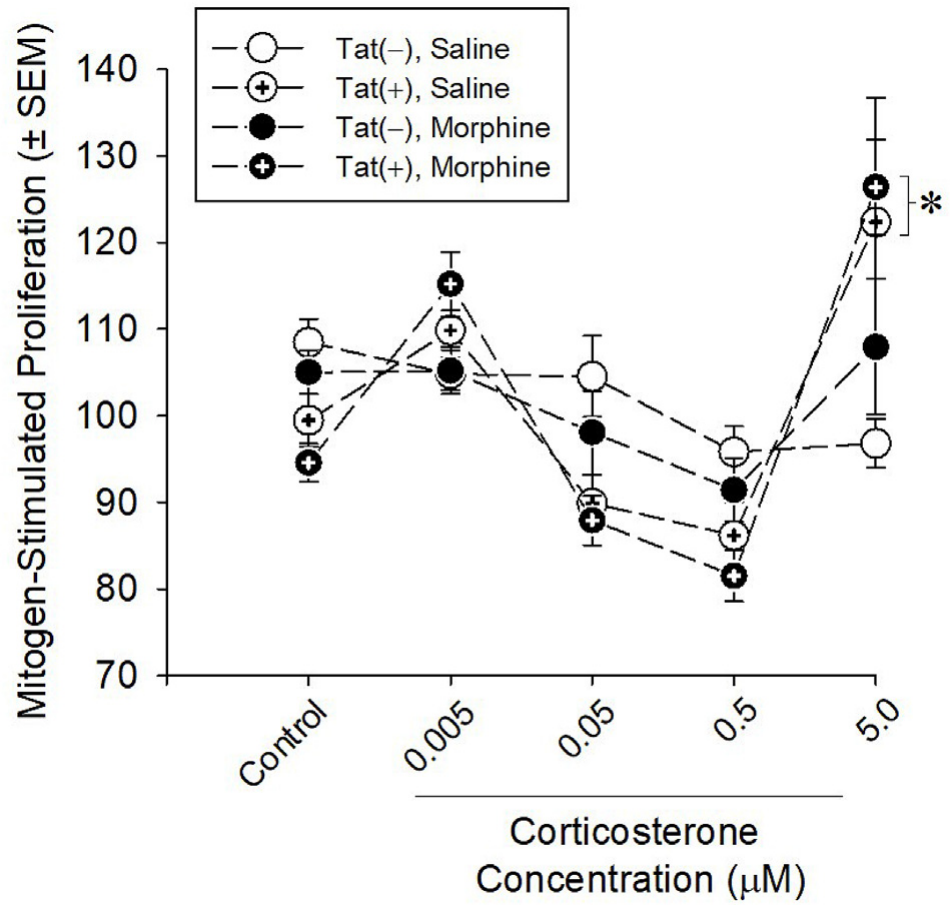
HIV-1 Tat-transgenic mice [Tat(+)] or their non-Tat-expressing counterparts [Tat(−)] were treated with saline or ramping morphine (10–40 mg/kg, s.c.) twice daily for 2 weeks and splenocytes were harvested and cultured (n = 6/group). Corticosterone (0.005, 0.05, 0.1, 0.5, or 5 μM) mediated inhibition of LPS-stimulated proliferation was used as an index of glucocorticoid resistance. * indicates a significant increase in Tat(+) splenocytes vs. Tat(−) splenocytes with 5 μM corticosterone (three-way ANOVA; *p* < 0.05).

Alterations in central and circulating steroid expression prompted further examination of the effects of HIV-1 Tat and morphine on peripheral glucocorticoid activity. Splenocytes were harvested from Tat(−) and Tat(+) mice after 2 weeks of ramping morphine or saline exposure and incubated with LPS and corticosterone for 48 h. Cell viability was used to assess the ability of corticosterone to suppress mitogen-stimulated splenocyte proliferation and survival. An inhibition of the ability of corticosterone to reduce splenocyte viability indicates glucocorticoid resistance. HIV-1 Tat significantly interacted with corticosterone concentration such that a greater proportion of Tat(+) splenocytes were resistant to 5 μM corticosterone-induced suppression of proliferation compared to Tat(−) splenocytes [*F*(4,25) = 10.24, *p* < 0.0001] (Fig. 3), indicating a resistance to the inhibitory effects of corticosterone. Tat did not significantly interact with morphine to influence glucocorticoid resistance.

**Fig. 4:**
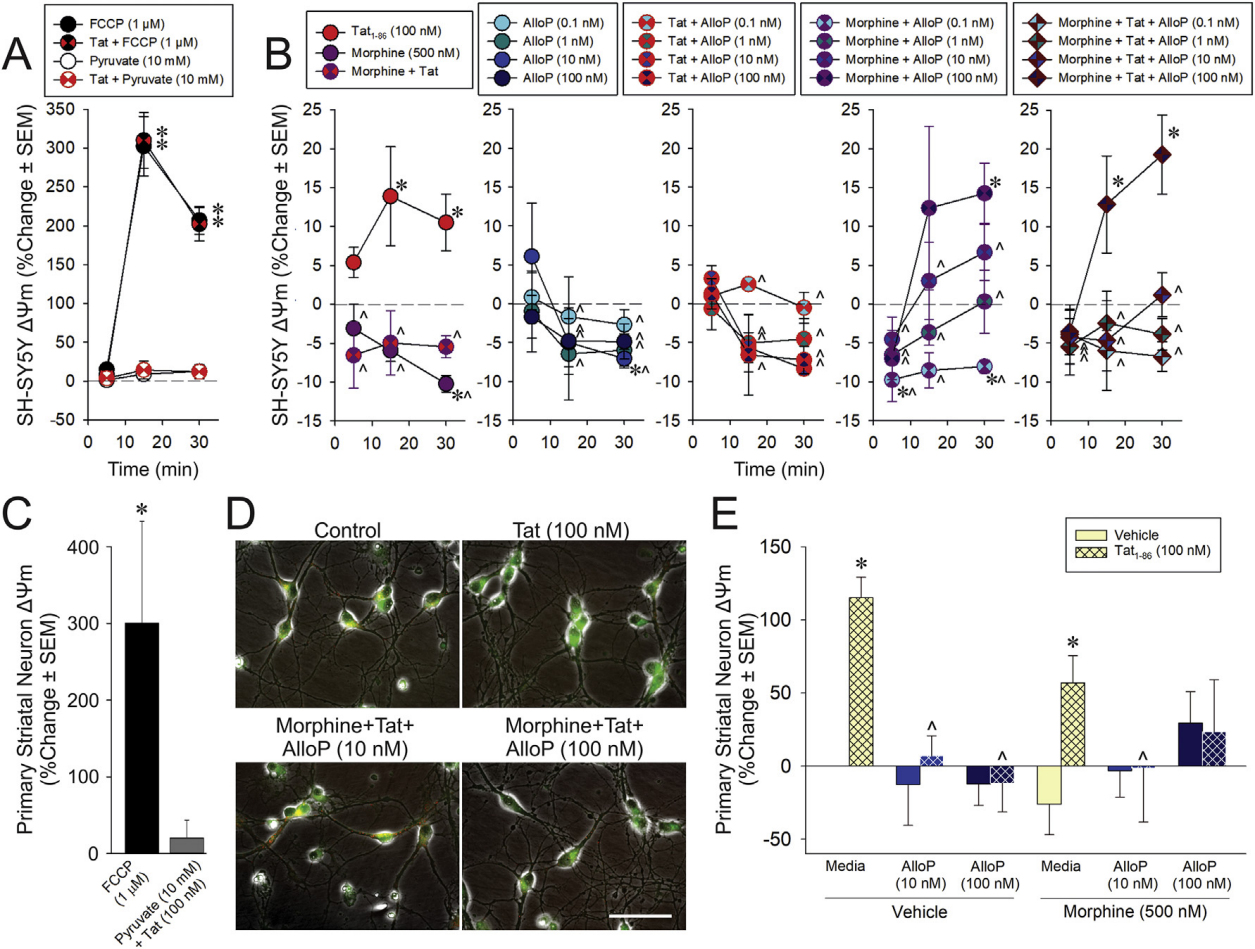
Mitochondrial membrane potential (Δψ_m_) of differentiated human neuroblastoma cells (SH-SY5Y; A-B) or C57BL/6J primary striatal medium spiny neurons (C-E; n = 4/group) depicted as a percent change from vehicle-treated control. (A) SH-SY5Y cells following positive (FCCP 1 μM) and negative (pyruvate 10 mM) control manipulations (with or without Tat 100 nM) or (B) treatment with Tat and/or morphine (500 nM) in combination with allopregnanolone (AlloP; 0.1, 1, 10, or 100 nM). Cells were assessed 5, 15, or 30 min following treatment. (C) C57BL/6J medium spiny neurons following positive (FCCP) and negative (pyruvate + Tat) control manipulations or (D,E) treatment with Tat and/or morphine in combination with AlloP (10, or 100 nM; bar indicates 50 μm). * indicates a significant difference from vehicle control (dashed line in panel B or solid line in panel E); ^∧^ indicates a significant difference from Tat-treated cells at the respective timepoint (open red circles in panel B or hatched bars in panel E); **indicates a significant difference from all other datapoints; (repeated-measures ANOVA in panels A,B; Student's *t*-test in panel C; three-way ANOVA in panel E; *p* < 0.05). (For interpretation of the references to colour in this figure legend, the reader is referred to the Web version of this article.)

### 3.4 Physiological, but not high, AlloP concentrations rescue Tat/morphine-dysregulated Δψ_m_

Given that alterations in central and circulating steroid synthesis were observed, we next assessed the influence of HIV-1 Tat and morphine on mitochondria which are known targets for Tat-mediated dysfunction and a rate-limiting source of steroidogenesis. We assessed the influence of Tat (100 nM) and/or morphine (500 nM) on mitochondrial membrane potential (Δψ_m_) via JC-10 using differentiated human neuroblastoma cells (SH-SY5Y) and primary, striatal, medium spiny neurons obtained from C57BL/6J mice. Central elevations of 5α-reduced progestogens, such as those seen here in response to Tat or morphine, are observed in models of brain injury and are proposed to underlie an endogenous mechanism of neural protection (Schumacher et al., 2014; Zhu et al., 2017). As such, we further assessed the potentially protective role that 5α-reduced pregnane steroids may play on Δψ_m_ following a Tat/morphine challenge by pretreating cells with a concentration-curve of AlloP (0–100 nM).

As respective positive and negative controls, cells were treated with the mitochondrial uncoupler, FCCP (1 μM), or pyruvate (10 mM) prior to vehicle or HIV-1 Tat exposure. As expected, FCCP caused significant depolarization of Δψ_m_ in differentiated SH-SY5Y cells [*F*(6,24) = 7.79, *p* = 0.0001] (Fig. 4A) or of C57BL/6J primary neurons [*F*(2,12) = 4.63, *p* = 0.03] (Fig. 4C). In SH-SY5Y cells, this effect was observed 15- or 30-min post-treatment and was not influenced by Tat (significant at each timepoint; *p* < 0.0001) indicating the cells were viable throughout the testing period. Adding pyruvate maintained Δψ_m_ in SH-SY5Y cells (Fig. 4A) and primary neurons (Fig. 4C) following acute exposure to Tat.

HIV-1 Tat, morphine, and/or AlloP exposure caused significant, time-dependent alterations in Δψ_m_ [*F*(38,120) = 1.84, *p* = 0.007] in differentiated SH-SY5Y cells (Fig. 4B). Compared to controls, HIV-1 Tat significantly depolarized Δψ_m_ within 15- and 30-min following treatment (*p* = 0.03–0.007; Fig. 4B). On its own, morphine significantly hyperpolarized Δψ_m_ 30 min post-treatment and significantly differed from Tat at every timepoint (*p* < 0.001–0.01; Fig. 4B). Morphine prevented Tat-induced Δψ_m_ depolarization at all timepoints assessed (*p* < 0.0001–0.004; Fig. 4B). At all but the highest concentration, AlloP did not affect Δψ_m_ when administered alone (*p* < 0.0001–0.02). The highest AlloP concentration (100 nM) significantly hyperpolarized Δψ_m_ at 30 min post-treatment (*p* = 0.02; Fig. 4B). Any concentration of AlloP reversed the effects of HIV-1 Tat (*p* < 0.0001–0.01). Unexpectedly, morphine and AlloP interacted to dysregulate Δψ_m_. In combination with morphine, the lowest AlloP concentration (0.1 nM) hyperpolarized Δψ_m_ (5 and 30 min post-treatment, *p* = 0.02–0.03), whereas the highest AlloP concentration significantly depolarized Δψ_m_ (30 min post-treatment, *p* = 0.02; Fig. 4B) consistent with an inhibition of electron transfer due to Ca^2+^ dysregulation. When Tat was added to combined morphine and AlloP, Δψ_m_ dysregulation was more rapid with the highest concentration of AlloP significantly depolarizing Δψ_m_ by 15 and 30 min, compared to controls (*p* = 0.0009–0.02; Fig. 4B), indicating a shift in mitochondrial function from an active to an inactive state.

In C57BL/6J primary neurons, HIV-1 Tat significantly interacted with AlloP to influence Δψ_m_ [*F*(2,36) = 6.17, *p* = 0.005] (Fig. 4D and E). Tat significantly depolarized Δψ_m_ compared to controls (*p* = 0.009) and pretreatment with AlloP (10 or 100 nM) significantly attenuated this effect (*p* = 0.0003–0.002; Fig. 4D and E). AlloP also significantly interacted with morphine [*F*(2,36) = 3.28, *p* < 0.05] such that AlloP abolished Tat or morphine-mediated depolarization at 10 nM, but this protection was lost when the highest AlloP concentration (100 nM) was co-administered with morphine (*p* > 0.05; Fig. 4D and E). Morphine and Tat did not significantly interact on their own. As such, Tat's contribution to the collapse of Δψ_m_ can be ameliorated by low-to-high physiological concentrations of AlloP. Morphine did not appear to contribute to Tat-mediated effects on Δψ_m_ among isolated medium spiny neurons, but did interact with the highest concentration of AlloP to promote mitochondrial membrane depolarization.

### 3.5 Physiological, but not high, AlloP rescues Tat-induced cell death

The capacity for AlloP to ameliorate Tat-mediated depolarization of Δψ_m_ prompted further examination of AlloP's direct protective capacity on long-term neuron viability. Cells were incubated with treatments for 20 h (a previously-established timepoint at which PROG or AlloP ameliorated Tat-mediated cell death; Salahuddin et al., 2019; Paris et al., 2016). The direct neurotoxic effects of Tat (i.e., in the absence of glial contribution), the concentration-dependent protective effects of AlloP, and their interactions with morphine were sought.

**Fig. 5:**
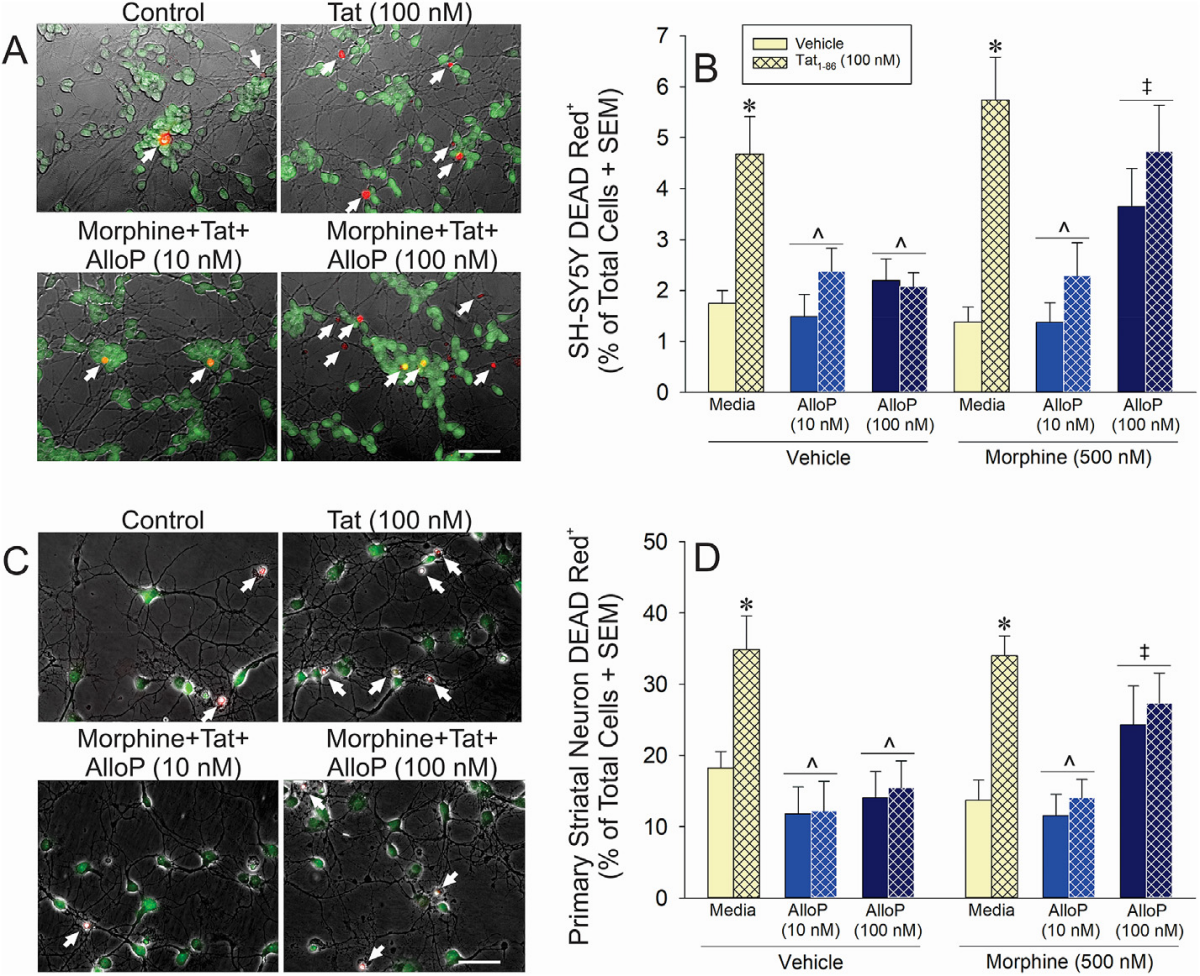
Live/dead assay of differentiated human neuroblastoma cells (SH-SY5Y; A-B; n = 3–5/group) or C57BL/6J primary striatal medium spiny neurons (C-D; n = 5–6/group) depicted as the proportion of DEAD Red^+^ cells following treatment with vehicle, morphine (500 nM), Tat (100 nM), and/or allopregnanolone (AlloP; 10 or 100 nM; bar indicates 50 μm). White arrows indicate necrotic cells. * indicates a significant difference from control; ^∧^ indicates a significant difference from Tat-treated cells; ‡ indicates a significant difference from Vehicle/AlloP (100 nM) treatment group (three-way ANOVA in panels B,D; *p* < 0.05). (For interpretation of the references to colour in this figure legend, the reader is referred to the Web version of this article.)

HIV-1 Tat significantly interacted with AlloP (10 or 100 nM) to influence neurotoxicity in both differentiated SH-SY5Y cells [*F*(2,33) = 9.38, *p* = 0.0006] (Fig. 5A and B) and C57BL/6J striatal medium spiny neurons [*F*(2,50) = 6.81, *p* = 0.002] (Fig. 5C and D). Tat significantly increased the proportion of necrotic cells compared to controls in either SH-SY5Y cells (*p* < 0.0001; Fig. 5A-B) or medium spiny neurons (*p* = 0.001; Fig. 5C-D) and AlloP (10 or 100 nM) significantly attenuated these effects (*p* = 0.004–0.01, Fig. 5B; *p* = 0.001–0.007, Fig. 5D). However, AlloP interacted with morphine in both SH-SY5Y cells [*F*(2,33) = 3.70, *p* = 0.04] and medium spiny neurons [*F*(2,50) = 3.70, *p* = 0.03] such that AlloP-mediated protection was lost when the highest concentration (100 nM) was co-administered with morphine (*p* > 0.05; Fig. 5B,D). Tat did not significantly interact with morphine to influence neurotoxicity. Thus, physiological AlloP ameliorated direct Tat-mediated neurotoxicity. High AlloP concentrations, such as those attained in the male rodent brain under stress, were neurotoxic in combination with a saturating concentration of morphine.

### 3.6 HIV-1 Tat potentiates, and AlloP attenuates, morphine's psychomotor effects

**Fig. 6:**
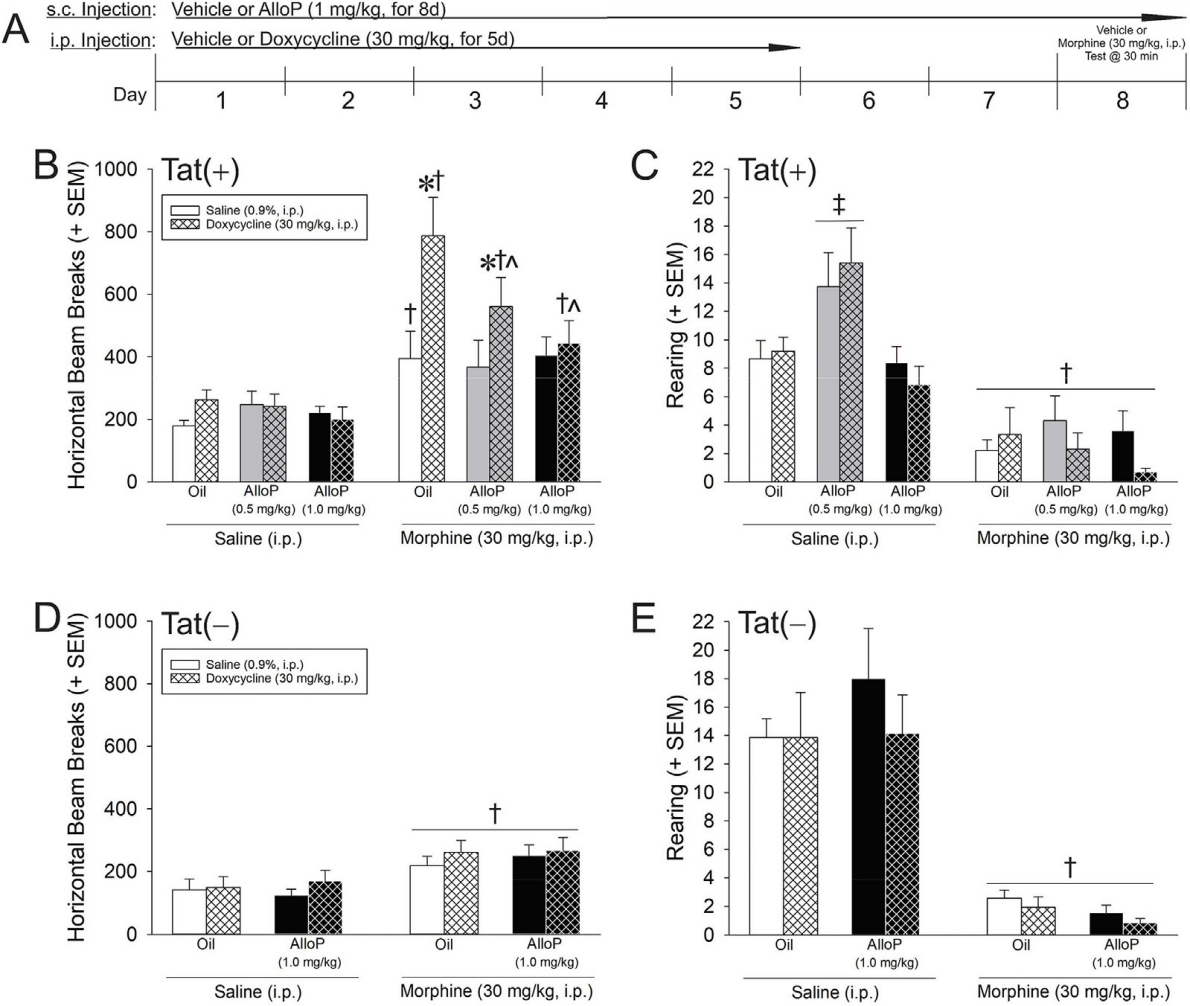
(A) HIV-1 Tat-transgenic mice (n = 14–16/group) were treated with vehicle or allopregnanolone (AlloP; 0.5 or 1.0 mg/kg, s.c.) for 8 days prior to testing and concurrent vehicle or doxycycline (30 mg/kg, i.p.) for 5 days prior to testing (with a 2-day doxycycline washout period prior to testing day). (B,D) Horizontal and (C,E) vertical (rearing) motor behavior were assessed 30 min after an acute injection of saline or morphine (30 mg/kg, i.p.) in Tat(+) and Tat(−) mice. * indicates a significant increase with Tat induction compared to respective uninduced control; † indicates a significant difference with morphine compared to respective saline-administered control; ^∧^ indicates a significant decrease compared to morphine/oil/Tat-induced mice; ‡ indicates a significant difference for saline/AlloP (0.5 mg/kg)-treated mice compared to all other groups (irrespective of Tat condition); (three-way ANOVA in panels B,C; *p* < 0.05).

To assess the functional behavioral interactions between HIV-1 Tat, opiates, and exogenous neurosteroid, Tat(+) mice were pretreated with vehicle or AlloP (0.5 or 1.0 mg/kg/d for 8 days, s.c.) while Tat was induced via doxycycline (30 mg/kg/d for 5 days, i.p.; allowing for 2 days of doxycycline-washout prior to the testing day). Multiple negative controls were included. To address the potential non-specific effects of doxycycline, some mice were administered saline (5 d, i.p.) as uninduced controls. To account for potential leaky transgene expression which can be observed in rtTA-transgenic mice, Tat(−) controls were commensurately treated using the high AlloP concentration (1.0 mg/kg). On day 8, mice received an acute dose of vehicle or morphine (30 mg/kg, i.p.) and were assessed for psychostimulated locomotion 30 min later (Fig. 6A).

Among Tat(+) mice, induction of HIV-1 Tat significantly interacted with administration of morphine [*F*(1,155) = 6.17, *p* = 0.01] and AlloP [*F*(2,155) = 3.25, *p* = 0.04] to influence horizontal locomotor activity (Fig. 6B). Morphine administration significantly increased locomotion in oil-administered controls (*p* < 0.0001–0.01; Fig. 6B). Inducing Tat significantly potentiated morphine's psychomotor effects compared to their uninduced counterparts (*p* < 0.0001). Pretreatment with AlloP (0.5 mg/kg) partly attenuated Tat-induced potentiation of morphine's locomotor effects (*p* = 0.02); however, this group still demonstrated greater motor behavior than did uninduced controls (*p* = 0.047). AlloP (1.0 mg/kg) fully attenuated Tat-induced potentiation of morphine's psychomotor effects, significantly differing from Tat-induced mice (*p* = 0.0005; Fig. 6B). Among Tat(−) control mice, morphine significantly increased locomotor behavior compared to saline [*F*(1,108) = 19.10, *p* < 0.001], but no effects of doxycycline or AlloP (1 mg/kg) were observed (Fig. 6D). Thus, Tat potentiated the psychomotor effect of acute morphine and this was ameliorated by exogenous AlloP (1 mg/kg).

Among Tat(+) mice, morphine and AlloP significantly interacted to influence rearing behavior, irrespective of Tat induction, [*F*(2,155) = 4.14, *p* = 0.02] (Fig. 6C). Following saline administration, mice that were pretreated with AlloP (0.5 mg/kg) reared significantly more than those pretreated with oil or AlloP (1.0 mg/kg; *p* = 0.001–0.006). Morphine significantly reduced rearing behavior in all Tat(+) groups (*p* < 0.0001). Tat(−) control mice also demonstrated a significant depression in rearing behavior following morphine administration, compared to that following saline [*F*(1,108) = 81.15, *p* < 0.001] (Fig. 6E). No effects of doxycycline or AlloP (1 mg/kg) were observed (Fig. 6E).

## 4 Discussion

The hypotheses that exposure to Tat and morphine would alter central or circulating steroid synthesis, directly promote neuronal mitochondrial dysfunction, and neuron cell death were partly upheld. Tat and morphine exerted separate effects to elevate steroid content, with Tat increasing central pregnenolone and 5α-reduced progestogens (while decreasing deoxycorticosterone) independent of changes in plasma. Morphine largely increased both central and circulating pregnane steroids, the glucocorticoid corticosterone, and their 5α-reduced metabolites consistent with activation of the HPA stress axis. In cell culture studies, Tat and morphine also exerted separate effects with Tat promoting glucocorticoid insensitivity, depolarizing Δψ_m_, and facilitating neuronal death, while morphine hyperpolarized Δψ_m_ and did not alter cell viability on its own. However, Tat and morphine did interact *in vivo*, with Tat potentiating the psychomotor effects of acute morphine in mice. Consistent with this, Tat exposure has been observed to potentiate acute or sensitized psychostimulant-induced locomotion and reward-related behavior of rodents (Harrod et al., 2008; Kesby et al., 2017; Mediouni et al., 2015a, Mediouni et al., 2015b; Paris et al., 2014a), as well as acute opioid-induced locomotion and behavioral disinhibition (Salahuddin et al., 2019). Notably, Tat's effects to potentiate psychostimulant-reward and opioid-mediated behavioral disinhibition could be attenuated concomitant with cyclical elevations in circulating hormones (Paris et al., 2014b; Salahuddin et al., 2019). The hypothesis that physiological concentrations of AlloP could ameliorate Tat-mediated neuronal insults and resulting changes in behavior was likewise upheld. However, an intriguing interaction was observed between high-concentration AlloP (100 nM) and morphine to depolarize Δψ_m_ and promote cell death. No untoward interactions between AlloP and morphine were observed *in vivo*; rather, AlloP (1.0 mg/kg) attenuated Tat's capacity to potentiate the physiological effects of acute morphine. These findings support the notion that central steroidogenesis is influenced by HIV-1 Tat or morphine and exogenous AlloP may exert several therapeutic benefits that are of interest for present and future work.

This study is the first to comprehensively profile central and circulating pregnane steroids in an animal model of neuroHIV. In other animal models of CNS insult, including ischemic stroke and traumatic brain injury, increased neurosteroidogenesis is observed and may reflect a local adaptive neuroprotective response (Guennoun et al., 2015; Liu et al., 2012; Schumacher et al., 2014; Zhu et al., 2017). In support, the 18 kDa translocator protein, which mediates mitochondrial cholesterol import for steroidogenesis, is highly expressed in microglia and astrocytes and is markedly upregulated following CNS injury and neuroinflammation (Rupprecht et al., 2010). Exogenous AlloP can improve experimental outcomes, including reductions to infarct volume, gliosis, cytokine upregulation, and improvements to post-recovery cognitive performance (Djebaili et al., 2005; Melcangi and Panzica, 2014; Sayeed et al., 2009). *In vitro*, AlloP is neuroprotective against hypoxic or excitotoxic insults (Frank and Sagratella, 2000). These effects are likely due in part to its pharmacodynamic profile as a potent positive allosteric modulator of GABA_A_ receptors [and direct agonist in high concentration (Callachan et al., 1987)] and as an antagonist of L-type Ca^2+^ channels (Earl and Tietz, 2011; Hu et al., 2007), readily restoring tonic inhibition within neural networks. As such, AlloP's protective effects against Tat may be partly due to its capacity to rapidly reinstate excitatory/inhibitory tone.

HIV-1 Tat and morphine independently altered steroidogenesis *in vivo*. In addition to local neurosteroidogenesis, Tat may influence central steroid biosynthesis via alterations in cholesterol homeostasis. RNA deep sequencing reveals Tat-mediated dysregulation of genes encoding for lipid and cholesterol homeostasis in rat hippocampal neurons (Mohseni Ahooyi et al., 2018). Moreover, exposure to Tat significantly increased free and total cholesterol and cholesteryl ester within cultured rat neurons, the latter of which was further increased by co-application of cocaine (Mohseni Ahooyi et al., 2018). Opposing effects for Tat (with or without cocaine) to reduce intra- and extracellular cholesterol in astrocytes have also been observed (Cotto et al., 2018), supporting the notion of dynamic changes in central cholesterol bioavailability following exposure to Tat and psychostimulants. Given that Tat increased splenocyte glucocorticoid resistance, it is reasonable to assume that the opioid-driven HPA response would be modified in an opioid-dependent organism.

Opiates are also demonstrated to influence steroidogenesis in rodents, albeit largely via HPA activation and with a bias towards synthesis of 5α-reduced metabolites, which can differ among species, throughout ontogeny, and depend on the nature and duration of opioid exposure (Bunce et al., 2015; Donegan and Bancos, 2018; Taylor et al., 1997). In support, acute or chronic morphine increases central and systemic 5α-reduction and AlloP formation in rodents (Concas et al., 2006; Porcu et al., 2014), effects that could be blocked by the 5α-reductase inhibitor, finasteride (Moradi-Azani et al., 2011; Verdi and Ahmadiani, 2007). While there are notable species-related differences in opioid activation of the HPA axis (Al-Hashimi et al., 2013; Vuong et al., 2010), morphine is observed to induce steroidogenesis in rodents via peripheral sources, particularly the adrenals (Concas et al., 2006; El Daly, 1996). Adrenally-derived corticosterone likely contributed to the great increase presently observed in the brain. High central glucocorticoid content is associated with neuronal injury and death (de Kloet et al., 2015; Sapolsky, 2015). The extent to which these effects may translate to opioid-dependent patients is not known; however, opioid dependence may reduce HPA responsivity and/or obviate diurnal ACTH/cortisol rhythmicity in people (Facchinetti et al., 1984; Vuong et al., 2010). Pharmacologically-inducing withdrawal in people can produce rebound HPA activation, adding complexity (Culpepper-Morgan et al., 1992, 1997; Kreek, 2007). When abused, the initially pleasurable effects of opioids dissipate and their continued use is largely motivated by a desire to escape the aversive effects of addiction (George et al., 2012; Koob, 2020; Koob and Kreek, 2007). Thus, while Tat-mediated changes in steroidogenesis may involve dysregulation of cholesterol homeostasis, interactions with opioid drugs of abuse may be expected to influence steroid-sensitive behaviors and are likely to differ with dependence vs. acute exposure.

HIV-1 Tat is known to disrupt mitochondrial function and maintaining AlloP at physiological levels may ameliorate mitochondrial deficits. Congruent with the current findings, Tat can depolarize mitochondrial membranes, induce opening of the mitochondrial permeability transition pore (mPTP), drive reactive oxygen species formation, and reduce ATP [reviewed in (Fields and Ellis, 2019; Rozzi et al., 2017)]. Conversely, AlloP or synthetic neurosteroid analogues maintain Δψ_m_, improve mitochondrial oxygen consumption rate and glycolysis, reduce opening of the mPTP, and preserve ATP synthesis in response to insult (Grimm et al., 2014; Lejri et al., 2017; Leśkiewicz et al., 2008a, Leśkiewicz et al., 2008b; Sayeed et al., 2009; Waters et al., 1997). However, protective effects of AlloP are not seen at high, pharmacological concentrations (Leśkiewicz et al., 2008a, Leśkiewicz et al., 2008b; Sayeed et al., 2009). On its own, morphine hyperpolarized Δψ_m_ which is consistent with past observations in primary mouse neurons (Fitting et al., 2014b) and with reduced mPTP opening and Cyt-*c* release in rat heart tissue (Chen et al., 2016). Physiological AlloP concentrations (e.g., 10 nM) may restore cellular and mitochondrial homeostasis. Thus, increased Tat-induced neurosteroidogenesis may be part of an adaptative cerebroprotective response, perhaps via the enhancement of AlloP concentrations in the 10 nM range that are mito- and neuroprotective. On the contrary, the large increases of corticosterone and AlloP (≂ 50 nM) that occur with morphine treatment may contribute to mitochondrial and neuronal dysfunction.

The mito/neurotoxic interactions between morphine and high-concentration AlloP (100 nM) are intriguing. In low-to-physiological concentrations, AlloP mediates phasic inhibition via activation of synaptic GABA_A_ Cl^−^ channels; at high concentrations, AlloP activates δ-subunit-containing, extrasynaptic GABA_A_ receptors mediating tonic or continuing Cl^−^ conductance [reviewed in (Reddy and Estes, 2016)]. In response to an excitotoxin, such as Tat, increased phasic or tonic Cl^−^ influx may be beneficial, restoring ion homeostasis. However, morphine-dependence is demonstrated to downregulate KCC2 in several CNS cell types leading to increased intracellular Cl^−^ concentrations (Ferrini et al., 2013; Gagnon et al., 2013; Taylor et al., 2016), which may promote Cl^−^ efflux in response to a potent agonist, such as AlloP. Moreover, AlloP exhibits high potency and is a particularly efficacious positive allosteric modulator of δ-containing GABA_A_ receptors (Carver and Reddy, 2016). Under these circumstances, high or supra-physiological AlloP and morphine may increase cell excitability. Whether supra-physiological concentrations of AlloP would produce toxic interactions with morphine *in vivo* is not known; however, benzodiazepines represent another class of positive GABA_A_ allosteric modulators that can exacerbate respiratory depression and many of the pathophysiological effects of opioids seen clinically (Dowell et al., 2016; Jones et al., 2012; White and Irvine, 1999). Future work will further investigate the influence of opioid dependence on AlloP-mediated Tat protection.

It should be noted that toxic interactions between opioids and Tat largely depend on glial μ-opioid receptors (Fitting et al., 2014b; Zou et al., 2011) and interactions with chemokine receptors (Festa and Meucci, 2012; Kim et al., 2018). The present findings utilized isolated neuron cultures to assess the direct neurotoxic relationship that may exist between HIV-1 Tat and morphine, as well as the potential for AlloP rescue. However, AlloP is demonstrated to influence immune responding, quiescing activated microglia (Ahmad et al., 2005; Müller and Kerschbaum, 2006) and directly or indirectly downregulating cytokine production (Ghezzi et al., 2000); immune factors that play a critical role in the progression of HIV (Thaney and Kaul, 2018). It will be important to parse the influence of neurotoxic bystander effects in these systems in the future.

## 5 Conclusions

These findings demonstrate the capacity for HIV-1 Tat or morphine to influence central and circulating pregnane steroidogenesis. Administration of exogenous AlloP in physiological concentrations ameliorates Tat-mediated mitochondrial dysfunction and neurotoxicity, even in combination with morphine. In mice, Tat potentiates, and AlloP reverses, morphine's psychomotor effects. Thus, adjunctive therapeutics that can maintain or enhance pregnane steroidogenesis may be beneficial for neuroHIV symptomology among opioid-using and opioid-naïve populations.

## Funding and disclosure

This work was supported by funds from the National Institutes of Health: R00 DA039791 (JJP), a pilot project from award P30 GM122733 (JJP), F30 DA044875 (SK), R01 DA024461 (PEK), R01 DA034231 (PEK & KFH), R01 DA018633 (KFH), R01 DA033200 (KFH), R01 DA045588 (KFH), and K02 DA027374 (KFH). The authors declare no conflict of interest.

## CRediT authorship contribution statement

**Jason J. Paris:** Conceptualization, Funding acquisition, Data curation, Formal analysis, Writing - original draft, Writing - review & editing. **Philippe Liere:** Conceptualization, Data curation, Formal analysis, Writing - original draft, Writing - review & editing. **Sarah Kim:** Funding acquisition, Data curation. **Fakhri Mahdi:** Data curation. **Meagan E. Buchanan:** Data curation. **Sara R. Nass:** Data curation, Formal analysis, Writing - original draft, Writing - review & editing. **Alaa N. Qrareya:** Data curation. **Mohammed F. Salahuddin:** Data curation. **Antoine Pianos:** Data curation. **Neïké Fernandez:** Data curation. **Zia Shariat-Madar:** Writing - original draft, Writing - review & editing. **Pamela E. Knapp:** Conceptualization, Funding acquisition, Writing - original draft, Writing - review & editing. **Michael Schumacher:** Conceptualization, Data curation, Formal analysis, Writing - original draft, Writing - review & editing. **Kurt F. Hauser:** Conceptualization, Funding acquisition, Writing - original draft, Writing - review & editing.

## References

[bib1] Adams S.M., Aksenova M.V., Aksenov M.Y., Mactutus C.F., Booze R.M. (2010). ER-β mediates 17β-estradiol attenuation of HIV-1 Tat-induced apoptotic signaling. Synapse.

[bib2] Adams S.M., Aksenova M.V., Aksenov M.Y., Mactutus C.F., Booze R.M. (2012). Soy isoflavones genistein and daidzein exert anti-apoptotic actions via a selective ER-mediated mechanism in neurons following HIV-1 Tat(1-86) exposure. PloS One.

[bib3] Ahmad I., Lope-Piedrafita S., Bi X., Hicks C., Yao Y., Yu C., Chaitkin E., Howison C.M., Weberg L., Trouard T.P., Erickson R.P. (2005). Allopregnanolone treatment, both as a single injection or repetitively, delays demyelination and enhances survival of Niemann-Pick C mice. J. Neurosci. Res..

[bib4] Al-Hashimi M., Scott S.W., Thompson J.P., Lambert D.G. (2013). Opioids and immune modulation: more questions than answers. Br. J. Anaesth..

[bib5] Ardeshiri A., Kelley M.H., Korner I.P., Hurn P.D., Herson P.S. (2006). Mechanism of progesterone neuroprotection of rat cerebellar Purkinje cells following oxygen-glucose deprivation. Eur. J. Neurosci..

[bib6] Avitsur R., Stark J.L., Sheridan J.F. (2001). Social stress induces glucocorticoid resistance in subordinate animals. Horm. Behav..

[bib7] Bons J., Moreau L., Lefebvre H. (2013). Adrenal disorders in human immunodeficiency virus (HIV) infected patients. Ann. Endocrinol..

[bib8] Bruce-Keller A.J., Turchan-Cholewo J., Smart E.J., Geurin T., Chauhan A., Reid R., Xu R., Nath A., Knapp P.E., Hauser K.F. (2008). Morphine causes rapid increases in glial activation and neuronal injury in the striatum of inducible HIV-1 Tat transgenic mice. Glia.

[bib9] Bunce S.C., Harris J.D., Bixler E.O., Taylor M., Muelly E., Deneke E., Thompson K.W., Meyer R.E. (2015). Possible evidence for re-regulation of HPA axis and brain reward systems over time in treatment in prescription opioid-dependent patients. J. Addiction Med..

[bib10] Cai N.S., Cadet J.L. (2008). The combination of methamphetamine and of the HIV protein, Tat, induces death of the human neuroblastoma cell line. SH-SY5Y. Synapse..

[bib11] Cai Y., Yang L., Callen S., Buch S. (2016). Multiple faceted roles of cocaine in potentiation of HAND. Curr. HIV Res..

[bib12] Callachan H., Cottrell G., Hather N., Lambert J., Nooney J., Peters J. (1987). Modulation of the GABA(A) receptor by progesterone metabolites. Proc. R. Soc. Lond. Biol. Sci..

[bib13] Carver C.M., Reddy D.S. (2016). Neurosteroid structure-activity relationships for functional activation of extrasynaptic δGABA(A) receptors. J. Pharmacol. Exp. Therapeut..

[bib14] Chen Z., Zhang X., Liu Y., Liu Z. (2016). Morphine postconditioning protects against reperfusion injury via inhibiting JNK/p38 MAPK and mitochondrial permeability transition pores signaling pathways. Cell. Physiol. Biochem..

[bib15] Chopard C., Tong P.B.V., Tóth P., Schatz M., Yezid H., Debaisieux S., Mettling C., Gross A., Pugnière M., Tu A., Strub J.M., Mesnard J.M., Vitale N., Beaumelle B. (2018). Cyclophilin A enables specific HIV-1 Tat palmitoylation and accumulation in uninfected cells. Nat. Commun..

[bib16] Chrousos G.P., Zapanti E.D. (2014). Hypothalamic-pituitary-adrenal axis in HIV infection and disease. Endocrinol Metab. Clin. N. Am..

[bib17] Concas A., Sogliano C., Porcu P., Marra C., Brundu A., Biggio G. (2006). Neurosteroids in nicotine and morphine dependence. Psychopharmacology (Berlin).

[bib18] Constantinescu R., Constantinescu A.T., Reichmann H., Janetzky B. (2007). Neuronal differentiation and long-term culture of the human neuroblastoma line SH-SY5Y. J. Neural. Transm. Suppl..

[bib19] Cotto B., Natarajaseenivasan K., Ferrero K., Wesley L., Sayre M., Langford D. (2018). Cocaine and HIV-1 Tat disrupt cholesterol homeostasis in astrocytes: implications for HIV-associated neurocognitive disorders in cocaine user patients. Glia.

[bib20] Culpepper-Morgan J.A., Kreek M.J. (1997). Hypothalamic-pituitary-adrenal axis hypersensitivity to naloxone in opioid dependence: a case of naloxone induced withdrawal. Metabolism.

[bib21] Culpepper-Morgan J.A., Inturrisi C.E., Portenoy R.K., Foley K., Houde R.W., Marsh F., Kreek M.J. (1992). Treatment of opioid-induced constipation with oral naloxone: a pilot study. Clin. Pharmacol. Ther..

[bib22] de Kloet A.D., Krause E.G., Solomon M.B., Flak J.N., Scott K.A., Kim D.H., Myers B., Ulrich-Lai Y.M., Woods S.C., Seeley R.J., Herman J.P. (2015). Adipocyte glucocorticoid receptors mediate fat-to-brain signaling. Psychoneuroendocrinology.

[bib23] Djebaili M., Guo Q., Pettus E.H., Hoffman S.W., Stein D.G. (2005). The neurosteroids progesterone and allopregnanolone reduce cell death, gliosis, and functional deficits after traumatic brain injury in rats. J. Neurotrauma.

[bib24] Donegan D., Bancos I. (2018). Opioid-induced adrenal insufficiency. Mayo Clin. Proc..

[bib25] Dowell D., Haegerich T.M., Chou R. (2016). CDC guideline for prescribing opioids for chronic pain--United States, 2016. J. Am. Med. Assoc..

[bib26] Earl D.E., Tietz E.I. (2011). Inhibition of recombinant L-type voltage-gated calcium channels by positive allosteric modulators of GABAA receptors. J. Pharmacol. Exp. Therapeut..

[bib27] El Daly E.S. (1996). Influence of acute and chronic morphine or stadol on the secretion of adrenocorticotrophin and its hypothalamic releasing hormone in the rat. Life Sci..

[bib28] El-Hage N., Gurwell J.A., Singh I.N., Knapp P.E., Nath A., Hauser K.F. (2005). Synergistic increases in intracellular Ca^2+^, and the release of MCP-1, RANTES, and IL-6 by astrocytes treated with opiates and HIV-1 Tat. Glia.

[bib29] El-Hage N., Bruce-Keller A.J., Yakovleva T., Bazov I., Bakalkin G., Knapp P.E., Hauser K.F. (2008). Morphine exacerbates HIV-1 Tat-induced cytokine production in astrocytes through convergent effects on [Ca(2+)](i), NF-kappaB trafficking and transcription. PloS One.

[bib30] Encinas M., Iglesias M., Liu Y., Wang H., Muhaisen A., Ceña V., Gallego C., Comella J.X. (2000). Sequential treatment of SH-SY5Y cells with retinoic acid and brain-derived neurotrophic factor gives rise to fully differentiated, neurotrophic factor-dependent, human neuron-like cells. J. Neurochem..

[bib31] Eugenin E.A., King J.E., Nath A., Calderon T.M., Zukin R.S., Bennett M.V., Berman J.W. (2007). HIV-tat induces formation of an LRP-PSD-95- NMDAR-nNOS complex that promotes apoptosis in neurons and astrocytes. Proc. Natl. Acad. Sci. U. S. A..

[bib32] Facchinetti F., Grasso A., Petraglia F., Parrini D., Volpe A., Genazzani A.R. (1984). Impaired circadian rhythmicity of beta-lipotrophin, beta-endorphin and ACTH in heroin addicts. Acta Endocrinol..

[bib33] Ferrini F., Trang T., Mattioli T.A., Laffray S., Del'Guidice T., Lorenzo L.E., Castonguay A., Doyon N., Zhang W., Godin A.G., Mohr D., Beggs S., Vandal K., Beaulieu J.M., Cahill C.M., Salter M.W., De Koninck Y. (2013). Morphine hyperalgesia gated through microglia-mediated disruption of neuronal Cl⁻ homeostasis. Nat. Neurosci..

[bib34] Festa L., Meucci O. (2012). Effects of opiates and HIV proteins on neurons: the role of ferritin heavy chain and a potential for synergism. Curr. HIV Res..

[bib35] Fields J.A., Ellis R.J. (2019). HIV in the cART era and the mitochondrial: immune interface in the CNS. Int. Rev. Neurobiol..

[bib37] Fitting S., Knapp P.E., Zou S., Marks W.D., Bowers M.S., Akbarali H.I., Hauser K.F. (2014). Interactive HIV-1 Tat and morphine-induced synaptodendritic injury is triggered through focal disruptions in Na^+^ influx, mitochondrial instability, and Ca^2+^ overload. J. Neurosci..

[bib36] Fitting S., Zou S., El-Hage N., Suzuki M., Paris J.J., Schier C.J., Rodríguez J.W., Rodriguez M., Knapp P.E., Hauser K.F. (2014). Opiate addiction therapies and HIV-1 Tat: interactive effects on glial [Ca^2^⁺]_i_, oxyradical and neuroinflammatory chemokine production and correlative neurotoxicity. Curr. HIV Res..

[bib38] Frank C., Sagratella S. (2000). Neuroprotective effects of allopregnenolone on hippocampal irreversible neurotoxicity in vitro. Prog. Neuro-Psychopharmacol. Biol. Psychiatry.

[bib39] Freda P.U., Bilezikian J.P. (1999). The hypothalamus-pituitary-adrenal axis in HIV disease. AIDS Read..

[bib40] Gabriel K.I., Cunningham C.L., Finn D.A. (2004). Allopregnanolone does not influence ethanol-induced conditioned place preference in DBA/2J mice. Psychopharmacology (Berlin).

[bib41] Gagnon M., Bergeron M.J., Lavertu G., Castonguay A., Tripathy S., Bonin R.P., Perez-Sanchez J., Boudreau D., Wang B., Dumas L., Valade I., Bachand K., Jacob-Wagner M., Tardif C., Kianicka I., Isenring P., Attardo G., Coull J.A.M., De Koninck Y. (2013). Chloride extrusion enhancers as novel therapeutics for neurological diseases. Nat. Med..

[bib42] George M.M., Bhangoo A. (2013). Human immune deficiency virus (HIV) infection and the hypothalamic pituitary adrenal axis. Rev. Endocr. Metab. Disord..

[bib43] George O., Le Moal M., Koob G.F. (2012). Allostasis and addiction: role of the dopamine and corticotropin-releasing factor systems. Physiol. Behav..

[bib44] Ghezzi P., Di Santo E., Sacco S., Foddi C., Barbaccia M.L., Mennini T. (2000). Neurosteroid levels are increased in vivo after LPS treatment and negatively regulate LPS-induced TNF production. Eur. Cytokine Netw..

[bib45] Gomes A.C., Aragüés J.M., Guerra S., Fernandes J., Mascarenhas M.R. (2017). Hypogonadotropic hypogonadism in human immunodeficiency virus-infected men: uncommonly low testosterone levels. Endocrinol. Diabetes Metab. Case Rep..

[bib46] Grassi D., Bellini M.J., Acaz-Fonseca E., Panzica G., Garcia-Segura L.M. (2013). Estradiol and testosterone regulate arginine-vasopressin expression in SH-SY5Y human female neuroblastoma cells through estrogen receptors-α and -β. Endocrinology.

[bib47] Grimm A., Schmitt K., Lang U.E., Mensah-Nyagan A.G., Eckert A. (2014). Improvement of neuronal bioenergetics by neurosteroids: implications for age-related neurodegenerative disorders. Biochim. Biophys. Acta.

[bib48] Guennoun R., Labombarda F., Gonzalez Deniselle M.C., Liere P., De Nicola A.F., Schumacher M. (2015). Progesterone and allopregnanolone in the central nervous system: response to injury and implication for neuroprotection. J. Steroid Biochem. Mol. Biol..

[bib49] Harrod S.B., Mactutus C.F., Fitting S., Hasselrot U., Booze R.M. (2008). Intra-accumbal Tat1-72 alters acute and sensitized responses to cocaine. Pharmacol. Biochem. Behav..

[bib50] Hategan A., Bianchet M.A., Steiner J., Karnaukhova E., Masliah E., Fields A., Lee M.H., Dickens A.M., Haughey N., Dimitriadis E.K., Nath A. (2017). HIV Tat protein and amyloid-β peptide form multifibrillar structures that cause neurotoxicity. Nat. Struct. Mol. Biol..

[bib51] Haughey N.J., Nath A., Mattson M.P., Slevin J.T., Geiger J.D. (2001). HIV-1 Tat through phosphorylation of NMDA receptors potentiates glutamate excitotoxicity. J. Neurochem..

[bib52] Hauser K.F., El-Hage N., Buch S., Nath A., Tyor W.R., Bruce-Keller A.J., Knapp P.E. (2006). Impact of opiate-HIV-1 interactions on neurotoxic signaling. J. Neuroimmune Pharmacol..

[bib53] Hauser K.F., Fitting S., Dever S.M., Podhaizer E.M., Knapp P.E. (2012). Opiate drug use and the pathophysiology of neuroAIDS. Curr. HIV Res..

[bib54] Hong W., Nuwayhid S.J., Werling L.L. (2004). Modulation of bradykinin-induced calcium changes in SH-SY5Y cells by neurosteroids and sigma receptor ligands via a shared mechanism. Synapse.

[bib55] Hu A.Q., Wang Z.M., Lan D.M., Fu Y.M., Zhu Y.H., Dong Y., Zheng P. (2007). Inhibition of evoked glutamate release by neurosteroid allopregnanolone via inhibition of L-type calcium channels in rat medial prefrontal cortex. Neuropsychopharmacology.

[bib56] Hu G., Yao H., Chaudhuri A.D., Duan M., Yelamanchili S.V., Wen H., Cheney P.D., Fox H.S., Buch S. (2012). Exosome-mediated shuttling of microRNA-29 regulates HIV Tat and morphine-mediated neuronal dysfunction. Cell Death Dis..

[bib57] Irwin R.W., Brinton R.D. (2014). Allopregnanolone as regenerative therapeutic for Alzheimer's disease: translational development and clinical promise. Prog. Neurobiol..

[bib58] Irwin R.W., Solinsky C.M., Loya C.M., Salituro F.G., Rodgers K.E., Bauer G., Rogawski M.A., Brinton R.D. (2015). Allopregnanolone preclinical acute pharmacokinetic and pharmacodynamic studies to predict tolerability and efficacy for alzheimer's disease. PloS One.

[bib59] Jones J.D., Mogali S., Comer S.D. (2012). Polydrug abuse: a review of opioid and benzodiazepine combination use. Drug Alcohol Depend..

[bib60] Kendall S.L., Anderson C.F., Nath A., Turchan-Cholewo J., Land C.L., Mactutus C.F., Booze R.M. (2005). Gonadal steroids differentially modulate neurotoxicity of HIV and cocaine: testosterone and ICI 182,780 sensitive mechanism. BMC Neurosci..

[bib61] Kesby J.P., Najera J.A., Romoli B., Fang Y., Basova L., Birmingham A., Marcondes M.C.G., Dulcis D., Semenova S. (2017). HIV-1 TAT protein enhances sensitization to methamphetamine by affecting dopaminergic function. Brain Behav. Immun..

[bib62] Kim S., Hahn Y.K., Podhaizer E.M., McLane V.D., Zou S., Hauser K.F., Knapp P.E. (2018). A central role for glial CCR5 in directing the neuropathological interactions of HIV-1 Tat and opiates. J. Neuroinflammation.

[bib63] Kinsey S.G., Bailey M.T., Sheridan J.F., Padgett D.A., Avitsur R. (2007). Repeated social defeat causes increased anxiety-like behavior and alters splenocyte function in C57BL/6 and CD-1 mice. Brain Behav. Immun..

[bib64] Koob G.F. (2020). Neurobiology of opioid addiction: opponent process, hyperkatifeia, and negative reinforcement. Biol. Psychiatr..

[bib65] Koob G., Kreek M.J. (2007). Stress, dysregulation of drug reward pathways, and the transition to drug dependence. Am. J. Psychiatr..

[bib66] Kreek M.J. (2007). Opioids, dopamine, stress, and the addictions. Dialogues Clin. Neurosci..

[bib67] Krogh K.A., Wydeven N., Wickman K., Thayer S.A. (2014). HIV-1 protein Tat produces biphasic changes in NMDA-evoked increases in intracellular Ca^2+^ concentration via activation of Src kinase and nitric oxide signaling pathways. J. Neurochem..

[bib68] Kruman I.I., Nath A., Mattson M.P. (1998). HIV-1 protein Tat induces apoptosis of hippocampal neurons by a mechanism involving caspase activation, calcium overload, and oxidative stress. Exp. Neurol..

[bib69] Lachâtre M., Pasquet A., Ajana F., Soudan B., Lion G., Bocket L., Cornavin P., Senneville E., Boufassa F., Chéret A. (2017). HIV and hypogonadism: a new challenge for young-aged and middle-aged men on effective antiretroviral therapy. AIDS.

[bib70] Lecoeur H., Borgne-Sanchez A., Chaloin O., El-Khoury R., Brabant M., Langonné A., Porceddu M., Brière J.J., Buron N., Rebouillat D., Péchoux C., Deniaud A., Brenner C., Briand J.P., Muller S., Rustin P., Jacotot E. (2012). HIV-1 Tat protein directly induces mitochondrial membrane permeabilization and inactivates cytochrome *c* oxidase. Cell Death Dis..

[bib71] Lejri I., Grimm A., Miesch M., Geoffroy P., Eckert A., Mensah-Nyagan A.G. (2017). Allopregnanolone and its analog BR 297 rescue neuronal cells from oxidative stress-induced death through bioenergetic improvement. Biochim. Biophys. Acta (BBA) - Mol. Basis Dis..

[bib72] Leśkiewicz M., Jantas D., Budziszewska B., Lasoń W. (2008). Excitatory neurosteroids attenuate apoptotic and excitotoxic cell death in primary cortical neurons. J. Physiol. Pharmacol..

[bib73] Leśkiewicz M., Regulska M., Budziszewska B., Jantas D., Jaworska-Feil L., Basta-Kaim A., Kubera M., Lasoń W. (2008). Neurosteroids enhance the viability of staurosporine and doxorubicin treated differentiated human neuroblastoma SH-SY5Y cells. Pharmacol. Rep..

[bib74] Liere P., Akwa Y., Weill-Engerer S., Eychenne B., Pianos A., Robel P., Sjövall J., Schumacher M., Baulieu E.E. (2000). Validation of an analytical procedure to measure trace amounts of neurosteroids in brain tissue by gas chromatography-mass spectrometry. J. Chromatogr. B Biomed. Sci. Appl..

[bib75] Liere P., Pianos A., Eychenne B., Cambourg A., Liu S., Griffiths W., Schumacher M., Sjövall J., Baulieu E.E. (2004). Novel lipoidal derivatives of pregnenolone and dehydroepiandrosterone and absence of their sulfated counterparts in rodent brain. J. Lipid Res..

[bib76] Liu A., Margaill I., Zhang S., Labombarda F., Coqueran B., Delespierre B., Liere P., Marchand-Leroux C., O'Malley B.W., Lydon J.P., De Nicola A.F., Sitruk-Ware R., Mattern C., Plotkine M., Schumacher M., Guennoun R. (2012). Progesterone receptors: a key for neuroprotection in experimental stroke. Endocrinology.

[bib77] Lockhart E.M., Warner D.S., Pearlstein R.D., Penning D.H., Mehrabani S., Boustany R.M. (2002). Allopregnanolone attenuates N-methyl-D-aspartate-induced excitotoxicity and apoptosis in the human NT2 cell line in culture. Neurosci. Lett..

[bib78] Maingat F.G., Polyak M.J., Paul A.M., Vivithanaporn P., Noorbakhsh F., Ahboucha S., Baker G.B., Pearson K., Power C. (2013). Neurosteroid-mediated regulation of brain innate immunity in HIV/AIDS: DHEA-S suppresses neurovirulence. Faseb. J..

[bib79] Malik S., Khalique H., Buch S., Seth P. (2011). A growth factor attenuates HIV-1 Tat and morphine induced damage to human neurons: implication in HIV/AIDS-drug abuse cases. PloS One.

[bib80] Mastrantonio R., Cervelli M., Pietropaoli S., Mariottini P., Colasanti M., Persichini T. (2016). HIV-tat induces the Nrf2/ARE pathway through NMDA receptor-elicited spermine oxidase activation in human neuroblastoma cells. PloS One.

[bib83] Melcangi R.C., Panzica G.C. (2014). Allopregnanolone: state of the art. Prog. Neurobiol..

[bib81] Mediouni S., Jablonski J., Paris J.J., Clementz M.A., Thenin-Houssier S., McLaughlin J.P., Valente S.T. (2015). Didehydro-cortistatin A inhibits HIV-1 Tat mediated neuroinflammation and prevents potentiation of cocaine reward in Tat transgenic mice. Curr. HIV Res..

[bib82] Mediouni S., Marcondes M.C., Miller C., McLaughlin J.P., Valente S.T. (2015). The cross-talk of HIV-1 Tat and methamphetamine in HIV-associated neurocognitive disorders. Front. Microbiol..

[bib84] Melcangi R.C., Maggi R., Martini L. (1993). Testosterone and progesterone metabolism in the human neuroblastoma cell line SH-SY5Y. J. Steroid Biochem. Mol. Biol..

[bib85] Mirza F.S., Luthra P., Chirch L. (2018). Endocrinological aspects of HIV infection. J. Endocrinol. Invest..

[bib86] Mohseni Ahooyi T., Shekarabi M., Torkzaban B., Langford T.D., Burdo T.H., Gordon J., Datta P.K., Amini S., Khalili K. (2018). Dysregulation of neuronal cholesterol homeostasis upon exposure to HIV-1 tat and cocaine revealed by RNA-sequencing. Sci. Rep..

[bib87] Moradi-Azani M., Ahmadiani A., Amini H. (2011). Increase in formalin-induced tonic pain by 5alpha-reductase and aromatase inhibition in female rats. Pharmacol. Biochem. Behav..

[bib88] Müller E., Kerschbaum H.H. (2006). Progesterone and its metabolites 5-dihydroprogesterone and 5-3-tetrahydroprogesterone decrease LPS-induced NO release in the murine microgdial cell line, BV-2. Neuroendocrinol. Lett..

[bib89] Murillo J.R., Goto-Silva L., Sánchez A., Nogueira F.C.S., Domont G.B., Junqueira M. (2017). Quantitative proteomic analysis identifies proteins and pathways related to neuronal development in differentiated SH-SY5Y neuroblastoma cells. EuPA Open Proteom..

[bib90] Napier T.C., Chen L., Kashanchi F., Hu X.T. (2014). Repeated cocaine treatment enhances HIV-1 Tat-induced cortical excitability via over-activation of L-type calcium channels. J. Neuroimmune Pharmacol..

[bib91] Nath A. (2015). Eradication of human immunodeficiency virus from brain reservoirs. J. Neurovirol..

[bib92] Nath A., Conant K., Chen P., Scott C., Major E.O. (1999). Transient exposure to HIV-1 Tat protein results in cytokine production in macrophages and astrocytes: a hit and run phenomenon. J. Biol. Chem..

[bib93] Nath A., Hauser K.F., Wojna V., Booze R.M., Maragos W., Prendergast M., Cass W., Turchan J.T. (2002). Molecular basis for interactions of HIV and drugs of abuse. J. Acquir. Immune Defic. Syndr..

[bib94] Paris J.J., Carey A.N., Shay C.F., Gomes S.M., He J.J., McLaughlin J.P. (2014). Effects of conditional central expression of HIV-1 tat protein to potentiate cocaine-mediated psychostimulation and reward among male mice. Neuropsychopharmacology.

[bib96] Paris J.J., Fenwick J., McLaughlin J.P. (2014). Progesterone protects normative anxiety-like responding among ovariectomized female mice that conditionally express the HIV-1 regulatory protein, Tat, in the CNS. Horm. Behav..

[bib95] Paris J.J., Fenwick J., McLaughlin J.P. (2014). Estrous cycle and HIV-1 Tat protein influence cocaine-conditioned place preference and induced locomotion of female mice. Curr. HIV Res..

[bib97] Paris J.J., Singh H.D., Ganno M.L., Jackson P., McLaughlin J.P. (2014). Anxiety-like behavior of mice produced by conditional central expression of the HIV-1 regulatory protein. Tat. Psychopharmacology (Berl).

[bib98] Paris J.J., Zou S., Hahn Y.K., Knapp P.E., Hauser K.F. (2016). 5α-reduced progestogens ameliorate mood-related behavioral pathology, neurotoxicity, and microgliosis associated with exposure to HIV-1 Tat. Brain Behav. Immun..

[bib99] Perry S.W., Barbieri J., Tong N., Polesskaya O., Pudasaini S., Stout A., Lu R., Kiebala M., Maggirwar S.B., Gelbard H.A. (2010). Human immunodeficiency virus-1 Tat activates calpain proteases via the ryanodine receptor to enhance surface dopamine transporter levels and increase transporter-specific uptake and Vmax. J. Neurosci..

[bib100] Porcu P., Locci A., Santoru F., Berretti R., Morrow A.L., Concas A. (2014). Failure of acute ethanol administration to alter cerebrocortical and hippocampal allopregnanolone levels in C57BL/6J and DBA/2J mice. Alcohol Clin. Exp. Res..

[bib101] Reddy D.S., Estes W.A. (2016). Clinical potential of neurosteroids for CNS disorders. Trends Pharmacol. Sci..

[bib102] Reddy D.S., Kulkarni S.K. (1996). Role of GABA-A and mitochondrial diazepam binding inhibitor receptors in the anti-stress activity of neurosteroids in mice. Psychopharmacology (Berlin).

[bib103] Rochira V., Guaraldi G. (2014). Hypogonadism in the HIV-infected man. Endocrinol Metab. Clin. N. Am..

[bib104] Rozzi S.J., Avdoshina V., Fields J.A., Trejo M., Ton H.T., Ahern G.P., Mocchetti I. (2017). Human immunodeficiency virus promotes mitochondrial toxicity. Neurotox. Res..

[bib105] Rupprecht R., Papadopoulos V., Rammes G., Baghai T.C., Fan J., Akula N., Groyer G., Adams D., Schumacher M. (2010). Translocator protein (18 kDa) (TSPO) as a therapeutic target for neurological and psychiatric disorders. Nat. Rev. Drug Discov..

[bib106] Saify K., Saadat M. (2016). Expression levels of OPRM1 and PDYN in human SH-SY5Y cells treated with morphine and methadone. Life Sci..

[bib107] Salahuddin M.F., Qrareya A.N., Mahdi F., Jackson D., Foster M., Vujanovic T., Box J.G., Paris J.J. (2019). Combined HIV-1 Tat and oxycodone activate the hypothalamic-pituitary-adrenal and -gonadal axes and promote psychomotor, affective, and cognitive dysfunction in female mice. Horm. Behav..

[bib108] Sapolsky R.M. (2015). Stress and the brain: individual variability and the inverted-U. Nat. Neurosci..

[bib109] Sayeed I., Parvez S., Wali B., Siemen D., Stein D.G. (2009). Direct inhibition of the mitochondrial permeability transition pore: a possible mechanism for better neuroprotective effects of allopregnanolone over progesterone. Brain Res..

[bib110] Schier C.J., Marks W.D., Paris J.J., Barbour A.J., McLane V.D., Maragos W.F., McQuiston A.R., Knapp P.E., Hauser K.F. (2017). Selective vulnerability of striatal D2 versus D1 dopamine receptor-expressing medium spiny neurons in HIV-1 Tat transgenic male mice. J. Neurosci..

[bib111] Schumacher M., Mattern C., Ghoumari A., Oudinet J.P., Liere P., Labombarda F., Sitruk-Ware R., De Nicola A.F., Guennoun R. (2014). Revisiting the roles of progesterone and allopregnanolone in the nervous system: resurgence of the progesterone receptors. Prog. Neurobiol..

[bib112] Shipley M.M., Mangold C.A., Szpara M.L. (2016). Differentiation of the SH-SY5Y human neuroblastoma cell line. J. Vis. Exp..

[bib113] Silverstein P.S., Shah A., Weemhoff J., Kumar S., Singh D.P., Kumar A. (2012). HIV-1 gp120 and drugs of abuse: interactions in the central nervous system. Curr. HIV Res..

[bib114] Singh I.N., Goody R.J., Dean C., Ahmad N.M., Lutz S.E., Knapp P.E., Nath A., Hauser K.F. (2004). Apoptotic death of striatal neurons induced by human immunodeficiency virus-1 Tat and gp120: differential involvement of caspase-3 and endonuclease G. J. Neurovirol..

[bib115] Soontornniyomkij V., Kesby J.P., Morgan E.E., Bischoff-Grethe A., Minassian A., Brown G.G., Grant I., Translational Methamphetamine AIDS Research Center (TMARC) Group (2016). Effects of HIV and methamphetamine on brain and behavior: evidence from human studies and animal models. J. Neuroimmune Pharmacol..

[bib116] Spector C., Mele A.R., Wigdahl B., Nonnemacher M.R. (2019). Genetic variation and function of the HIV-1 Tat protein. Med. Microbiol. Immunol..

[bib117] Sun X., Shi X., Lu L., Jiang Y., Liu B. (2016). Stimulus-dependent neuronal cell responses in SH-SY5Y neuroblastoma cells. Mol. Med. Rep..

[bib118] Taylor C.C., Soong Y.I., Wu D., Yee J.S., Szeto H.H. (1997). Morphine stimulates adrenocorticotropin and cortisol release in the late-term ovine fetus. Pediatr. Res..

[bib119] Taylor A.M.W., Castonguay A., Ghogha A., Vayssiere P., Pradhan A.A., Xue L., Mehrabani S., Wu J., Levitt P., Olmstead M.C., De Koninck Y., Evans C.J., Cahill C.M. (2016). Neuroimmune regulation of GABAergic neurons within the ventral tegmental area during withdrawal from chronic morphine. Neuropsychopharmacology.

[bib120] Thaney V.E., Kaul M. (2018). Type I interferons in NeuroHIV. Viral Immunol..

[bib121] Toll L., Polgar W.E., Auh J.S. (1997). Characterization of the delta-opioid receptor found in SH-SY5Y neuroblastoma cells. Eur. J. Pharmacol..

[bib122] Verdi J., Ahmadiani A. (2007). Finasteride, a 5α-reductase inhibitor, potentiates antinociceptive effects of morphine, prevents the development of morphine tolerance and attenuates abstinence behavior in the rat. Horm. Behav..

[bib123] Vuong C., Van Uum S.H., O'Dell L.E., Lutfy K., Friedman T.C. (2010). The effects of opioids and opioid analogs on animal and human endocrine systems. Endocr. Rev..

[bib124] Wallace D.R., Dodson S., Nath A., Booze R.M. (2006). Estrogen attenuates gp120- and tat1-72-induced oxidative stress and prevents loss of dopamine transporter function. Synapse.

[bib125] Waters S.L., Miller G.W., Aleo M.D., Schnellmann R.G. (1997). Neurosteroid inhibition of cell death. Am. J. Physiol..

[bib126] White J.M., Irvine R.J. (1999). Mechanisms of fatal opioid overdose. Addiction.

[bib127] Wong N., Levy M., Stephenson I. (2017). Hypogonadism in the HIV-infected man. Curr. Treat. Options Infect. Dis..

[bib128] Zhu X., Yao H., Peng F., Callen S., Buch S. (2009). PDGF-mediated protection of SH-SY5Y cells against Tat toxin involves regulation of extracellular glutamate and intracellular calcium. Toxicol. Appl. Pharmacol..

[bib129] Zhu X., Fréchou M., Liere P., Zhang S., Pianos A., Fernandez N., Denier C., Mattern C., Schumacher M., Guennoun R. (2017). A role of endogenous progesterone in stroke cerebroprotection revealed by the neural-specific deletion of its intracellular receptors. J. Neurosci..

[bib130] Zou S., Fitting S., Hahn Y.K., Welch S.P., El-Hage N., Hauser K.F., Knapp P.E. (2011). Morphine potentiates neurodegenerative effects of HIV-1 Tat through actions at μ-opioid receptor-expressing glia. Brain.

